# Ion channel profiling of the *Lymnaea stagnalis* ganglia via transcriptome analysis

**DOI:** 10.1186/s12864-020-07287-2

**Published:** 2021-01-06

**Authors:** Nancy Dong, Julia Bandura, Zhaolei Zhang, Yan Wang, Karine Labadie, Benjamin Noel, Angus Davison, Joris M. Koene, Hong-Shuo Sun, Marie-Agnès Coutellec, Zhong-Ping Feng

**Affiliations:** 1 Department of Physiology, University of Toronto, 3308 MSB, 1 King’s College Circle, Toronto, ON M5S 1A8 Canada; 2 Donnelly Centre for Cellular and Biomolecular Research and Department of Molecular Genetics, University of Toronto, Toronto, ON M5S 3E1 Canada; 3 Department of Ecology and Evolutionary Biology, University of Toronto, Toronto, Ontario M5S 3B2 Canada; 4 Department of Biological Sciences, University of Toronto Scarborough, Toronto, Ontario M1C 1A4 Canada; 5 Genoscope, Institut de biologie François Jacob, Commissariat à lʼEnergie Atomique (CEA), Université Paris-Saclay, BP5706, 91057 Evry, France; 6 Génomique Métabolique, Genoscope, Institut François Jacob, CEA, CNRS, University of Evry, Université Paris-Saclay, 91057 Evry, France; 7 School of Life Sciences, University of Nottingham, University Park, Nottingham, UK, NG7 2RD UK; 8 Department of Ecological Science, Faculty of Science, Vrije Universiteit, Amsterdam, The Netherlands; 9 Department of Surgery, University of Toronto, Toronto, Ontario M5S 1A8 Canada; 10 ESE, Ecology and Ecosystem Health, INRAE, Agrocampus Ouest, 35042 Rennes, France

**Keywords:** *Lymnaea stagnalis*, Ion channels, Ionotropic receptors, Transcriptome, de novo assembly, CNS

## Abstract

**Background:**

The pond snail *Lymnaea stagnalis (L. stagnalis)* has been widely used as a model organism in neurobiology, ecotoxicology, and parasitology due to the relative simplicity of its central nervous system (CNS). However, its usefulness is restricted by a limited availability of transcriptome data. While sequence information for the *L. stagnalis* CNS transcripts has been obtained from EST libraries and a de novo RNA-seq assembly, the quality of these assemblies is limited by a combination of low coverage of EST libraries, the fragmented nature of de novo assemblies, and lack of reference genome.

**Results:**

In this study, taking advantage of the recent availability of a preliminary *L. stagnalis* genome, we generated an RNA-seq library from the adult *L. stagnalis* CNS, using a combination of genome-guided and de novo assembly programs to identify 17,832 protein-coding *L. stagnalis* transcripts. We combined our library with existing resources to produce a transcript set with greater sequence length, completeness, and diversity than previously available ones. Using our assembly and functional domain analysis, we profiled *L. stagnalis* CNS transcripts encoding ion channels and ionotropic receptors, which are key proteins for CNS function, and compared their sequences to other vertebrate and invertebrate model organisms. Interestingly, *L. stagnalis* transcripts encoding numerous putative Ca^2+^ channels showed the most sequence similarity to those of *Mus musculus*, *Danio rerio*, *Xenopus tropicalis*, *Drosophila melanogaster*, and *Caenorhabditis elegans*, suggesting that many calcium channel-related signaling pathways may be evolutionarily conserved.

**Conclusions:**

Our study provides the most thorough characterization to date of the *L. stagnalis* transcriptome and provides insights into differences between vertebrates and invertebrates in CNS transcript diversity, according to function and protein class. Furthermore, this study provides a complete characterization of the ion channels of *Lymnaea stagnalis*, opening new avenues for future research on fundamental neurobiological processes in this model system.

**Supplementary Information:**

The online version contains supplementary material available at 10.1186/s12864-020-07287-2.

## Background

The pond snail *Lymnaea stagnalis* is a widely used model organism in neurobiology, development, ecotoxicology, and parasitology. Its central nervous system (CNS) consists of large singly identifiable neurons and simple, well-characterized neural circuits, which allows detailed electrophysiological, biochemical, and molecular analyses of the cellular basis of behavior, including sensorimotor integration [1], learning and memory [2, 3], central pattern generator (CPG) networks [4], and neuromodulation [5]. In addition, *L. stagnalis* also exhibits notable traits absent in other orders of organisms, such as anoxia-tolerance [6] and central nervous system regeneration [7]. Elucidating the mechanisms underlying these traits may provide key insights into therapeutic strategies for humans. Also, as a widely-distributed organism in freshwater ecosystems all over the world, *L. stagnalis* has recently been adopted as the model species of a new OECD test guideline [8]. It has been used as a biomonitor for the effects of a variety of water conditions and pollutants, including heavy metals [9, 10], water acidification [11], and pesticide use [12, 13]. Finally, *L. stagnalis* is relatively closely related to the land snail *Biomphalaria glabrata*, a vector for *Schistosoma mansoni*, the parasite that is the cause for schistosomiasis [14], a prominent public health burden in developing countries. Infection by *S. mansoni* elicits significant changes in neuropeptide dynamics in the *B. glabrata* CNS [15]. Interestingly, *L. stagnalis* is shown to be resistant to *S. mansoni* infection [16], suggesting that it may provide clues into strategies for preventing *S. mansoni* transmission by *B. glabrata*. In each case, thorough molecular characterization of the *L. stagnalis* CNS is key to realizing the translational potential of this model organism.

As ion channels are the fundamental units of excitability and synaptic transmission in the nervous system, characterization and identification of ion channels is enormously important to the field of neuroscience. Mutations in the genes encoding ion channels, or encoding proteins that regulate ion channels, have dramatic effects on normal functioning, resulting in a wide range of debilitating diseases [17, 18]. Therefore, thorough molecular characterization of ion channels has enormous basic and translational impacts on our understanding of the nervous system and beyond, necessitating characterization of ion channels in newly sequenced transcriptomes of model organisms such as *L. stagnalis*.

Animals have evolved a broad diversity of ion channels. In mammals, approximately 80 genes encode potassium channel subunits, forming 4 families and 7 subfamilies of potassium channels [19–21]. Though sodium and calcium channels are less numerous, their functional diversity is also rich. The diversity of the 5 classes of voltage-gated calcium channels is conferred by different α, β, δ, γ subunit composition, while splice variation of those subunits gives rise to varied kinetics and pharmacology [22]. Furthermore, diversity of mammalian sodium channels is conferred by a variety of α- and β-subunits [23]. Finally, ligand-gated ion channels, though having considerably less variation, still vary in their permeabilities, kinetics, ligands, and subunit compositions [24]. They include ionotropic glutamate receptors [25], GABA_A_ receptors [26], nicotinic acetylcholine receptors [27], ionotropic serotonin 5-HT3 receptors [28], and purinergic receptors [29].

Though the kinetics, pharmacology, permeability, and conductivity of the various ion channels are diverse, many ion channels are widely evolutionarily conserved. One of the earliest potassium channels to be characterized in *Drosophila*, the Shaker K+ channel, now known as Kv1.3, shares 82% sequence homology with the rat homologue [30]. This refrain of invertebrate channels being conserved in mammals is repeated in the mouse homologues of *Drosophila* Shaker, Shal, Shab, and Shaw K^+^ channels, and in humans as well, with the *Drosophila* K+ channels all having been shown to be expressed in human cardiac tissue [31]. On the molecular level, the segments of potassium channels that are evolutionarily conserved across vertebrates and invertebrates are those that are most important for channel function: the voltage gate, the selectivity filter, and the segment of the pore that gives the pore its shape [32]. This indicates that evolutionarily conserved ion channels are essential for fundamental processes in neurobiology.

The first ion channel to be cloned from *L. stagnalis* was the GABA_A_ receptor, an ionotropic receptor and ligand-gated ion channel [33]. Since then, other evolutionarily conserved ion channels have been cloned from *L. stagnalis*, including glutamate receptor subunits [34–36], acetylcholine receptor [37], L-type, P/Q-type, N-type, R-type voltage-gated calcium channels and NALCN-like sodium leak channel [38–40], T-type voltage-gated calcium channels [41], and P2X receptor [42]. Fellow mollusc *Aplysia californica*, an immensely important model organism for neuroscience, has been used extensively to study ion channels [43]. Study of its neuronal transcriptome has revealed expression of a variety of ion channels, including ionotropic glutamate, acetylcholine, and GABA/glycine receptors, voltage-gated Ca^2+^ and Na^+^ channels, several families of K^+^ channels, amiloride-sensitive Na^+^ channels, cyclic nucleotide-gated channels, and inositol trisphosphate/ryanodine receptors [44]. Another molluscan model organism, *Biomphalaria glabrata*, has been shown to express transcripts encoding various ion channels as well, including Na^+^, K^+^, Cl^−^, Ca^2+^, and TRP cation channels, as well as glutamate, acetylcholine, and GABA receptors [45]. Furthermore, novel, evolutionarily conserved ion channels have been first identified and characterized in various invertebrate species. For instance, the first hyperpolarization-activated cyclic nucleotide-gated (HCN) channel was cloned from sea urchin sperm [46], leading to the discovery of several mammalian homologues. Furthermore, *D. melanogaster* has been used to identify the molecular determinants of the newest family of ion channels, calcium release-activated current (CRAC) channels [47, 48]. This demonstrates that invertebrate models, including *L. stagnalis*, continue to be a rich resource of discovery for evolutionarily conserved fundamental neuronal signaling processes applicable to all organisms.

Recent advances in high-throughput RNA sequencing (RNAseq) have much accelerated the search for novel ion channel and receptor families. Current sequence information for the *L. stagnalis* CNS is obtained from EST libraries [49] and a de novo RNA-seq assemblies [50]. However, due to the low coverage of EST libraries and fragmented nature of de novo assemblies, much remains to be characterized about the *L. stagnalis* CNS transcriptome. The recent availability of a preliminary *L. stagnalis* reference genome [51] has provided the opportunity to enhance the completeness and coverage of the CNS transcriptome through genome-guided assembly of RNA-seq reads. This can potentially allow identification of novel *L. stagnalis* transcripts and characterization of whole protein classes, particularly of ion channels and ionotropic receptors, which have not yet been fully characterized. In this study, we employed a combination of genome-guided and de novo assembly programs to create an assembly that improves upon the completeness and coverage of existing resources. We have also identified transcripts encoding ion channels and ionotropic receptors and compared them to other species. This provides the most thorough characterization to date of the CNS transcriptome of this important molluscan model organism and builds a crucial foundation for leveraging the unique advantages of *L. stagnalis* in a wide range of research fields. Furthermore, as this study characterizes ion channels in an invertebrate molluscan species, where essential neurobiological processes are likely to be evolutionarily conserved across species, this study serves as a potential starting point for the identification of novel ion channel families which may be evolutionarily conserved in mammals.

## Results

### CNS transcriptome assembly and annotation

**Fig. 1:**
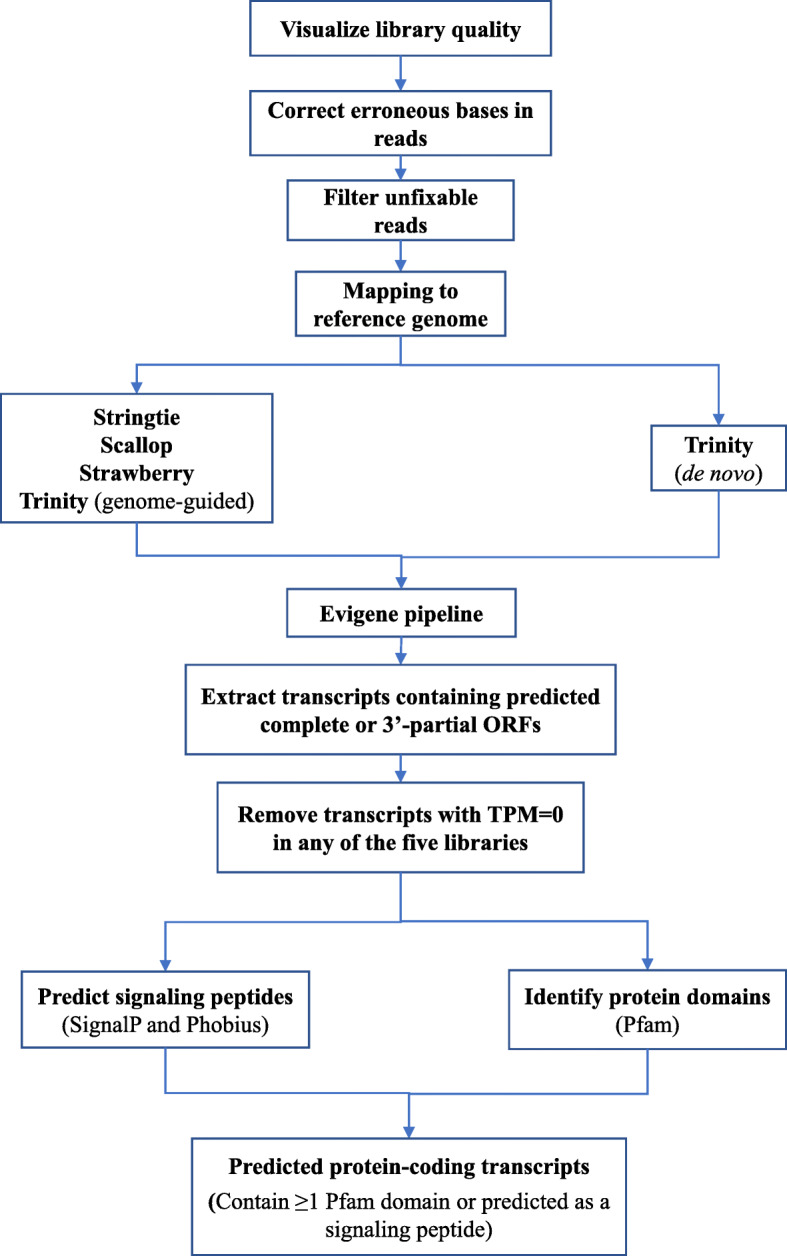
Workflow of quality control, assembly and prediction of protein-coding transcripts in the *L. stagnalis* CNS

*L. stagnalis* is a widely used model organism in understanding the fundamental mechanisms of neural function due to its simple and well-characterized CNS. A de novo assembly of the *L. stagnalis* CNS transcriptome from 100 bp single-end reads has previously been published [50], but the completeness of the assembly had been hindered by the lack of a genome reference. With the aid of a preliminary, recently sequenced and assembled *L. stagnalis* genome, here we employed a combination of genome-guided and de novo approaches to assemble reads from four 150 bp paired-end libraries that we prepared from four adult central ring ganglia and the aforementioned published 100 bp single-end library (Sadamoto et al. 2012) to create a new and improved *L. stagnalis* CNS transcriptome assembly (Fig. 1). After correcting for erroneous bases and removing unfixable reads (Table S3), each of the five libraries had at least 88% of the reads mapped to the genome with unique location (Table S4). The mapped reads in each library were assembled in a genome-guided manner using multiple assemblers and the unmapped reads were pooled for de novo assembly using Trinity. Altogether, this resulted in 22 assemblies containing 1,651,924 transcripts in total (Table S5). To identify putative protein-coding transcripts (Fig. 1), we analyzed this set of sequences using the Evigene pipeline to identify 196,514 non-redundant transcripts that contained a complete or 3′-partial (containing start but not stop codon) open reading frame (ORF) (Table S6). Potential transcript artifacts and noise were filtered out, resulting in a transcript set containing 68,094 transcripts. As ORFs can arise spuriously [52], we further identified protein domain-containing transcripts by annotation against the Pfam database and/or signaling peptide prediction by both SignalP and Phobius, resulting in 17,832 sequences as the final set of predicted protein-coding transcripts. Annotation of the top 20 most highly expressed predicted protein-coding transcripts showed that the majority were annotated or predicted as signaling peptides (Table 1).

**Table 1:** BLAST hit of the top 20 expressed transcripts in the *L. stagnalis* CNS

Accession	Gene name	Species	Identity (%)	Description
BAW32915.1	Cytochrome c oxidase subunit I, partial (mitochondrion)	*Ringicula* cf. *pilula*	84	Energy production
AAB29129.1	preproLYCP	*Lymnaea stagnalis*	99.1	
P42579.1	Sodium-influx-stimulating peptide	*Lymnaea stagnalis*	100	
Predicted signaling peptide				Neuropeptide signaling
P80090.2	Molluscan insulin-related peptide 3	*Lymnaea stagnalis*	99.2	
ABV22501.1	Myoglobin	*Biomphalaria tenagophila*	69.5	Oxygen transport
Predicted signaling peptide				Neuropeptide signaling
P58154.1	Acetylcholine-binding protein	*Lymnaea stagnalis*	98.3	Synaptic transmission
Predicted signaling peptide				Neuropeptide signaling
P42577.2	Soma ferritin	*Lymnaea stagnalis*	100	Metal ion homeostasis
YP_006665701.1	Cytochrome c oxidase subunit III (mitochondrion)	*Galba pervia*	85	Energy production
AAD02473.1	Cardioexcitatory peptide precursor	*Lymnaea stagnalis*	94.1	Neuropeptide signaling
AAS20460.1	Granularin	*Lymnaea stagnalis*	100	Defense response
Predicted signaling peptide				Neuropeptide signaling
P48416.1	Cytochrome P450	*Lymnaea stagnalis*	96.3	Oxidoreductase activity
XP_012943264.1	PREDICTED: uncharacterized protein LOC106013068	*Aplysia californica*	45.2	
P06308.2	Ovulation prohormone	*Lymnaea stagnalis*	87.4	
Predicted signaling peptide				Neuropeptide signaling
ARS01367.1	Ffamide 2	*Deroceras reticulatum*	58.2	
Predicted signaling peptide				

### Comparison and aggregation of current assembly with previously published *L. stagnalis* CNS transcriptome

To compare the number and quality of predicted protein-coding transcripts uncovered in our current assembly with those in the previously published expressed sequence tags (EST) library [49] and de novo assembly [50], these two earlier sets of sequences were processed as described above to identify potential protein coding transcripts. We found 1946 sequences (out of 10,375 in total) that contained at least one protein domain and/or were annotated as a signaling peptide by both Phobius and SignalP in the EST library, and 11,742 such sequences (out of 116,355 in total) in the de novo assembled library. BUSCO analysis showed that the predicted protein-coding transcripts identified in the current assembly contained 92.7% of single-copy ortholog genes present in > 90% eukaryotic species, higher than the de novo assembly (91.5%) and the EST library (13.4%) (Table 2).

**Table 2:** BUSCO analyses of protein-coding sequences identified in the published EST library and de novo assembly, the current assembly and combined set

	Complete (single-copy)	Complete (duplicated)	Fragmented	Missing
Current assembly	**803** (82.1%)	**104** (10.6%)	**3** (0.3%)	**68** (7.0%)
De novo assembly	**885** (90.5%)	**10** (1.0%)	**13** (1.3%)	**70** (7.2%)
EST library	**124** (12.7%)	**7** (0.72%)	**61** (6.2%)	**786** (80.4%)
Combined	**907** (92.7%)	**34** (3.5%)	**3** (0.3%)	**34** (3.5%)

**Fig. 2:**
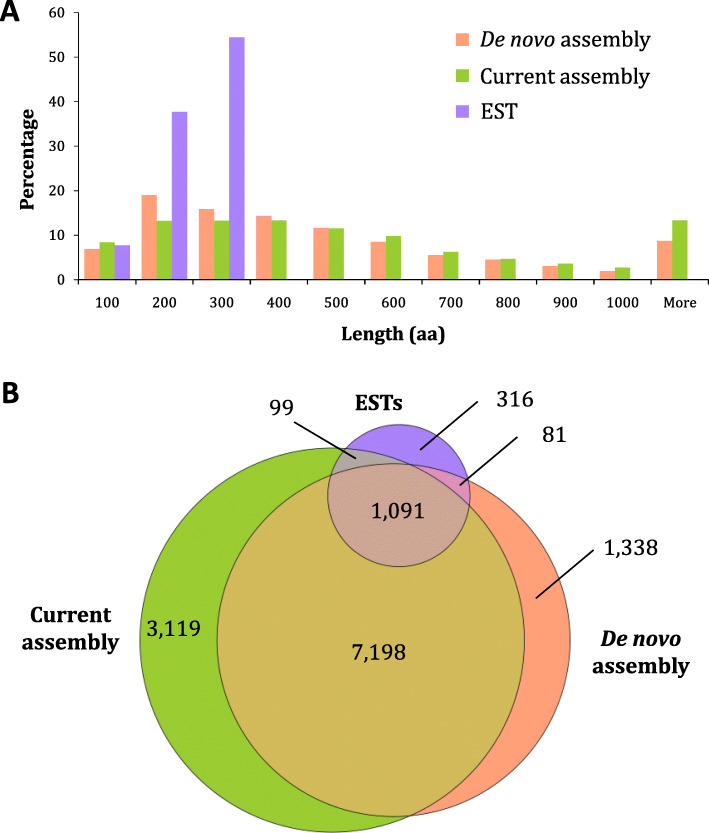
Comparison of predicted *L. stagnalis* protein-coding transcripts identified in the current assembly with those identified in the previously published EST library [49] and de novo assembly [50]. **a** Distribution of translated amino acid sequence lengths of predicted protein-coding transcripts as a percentage of the total number of transcripts in previous and current assemblies. The current assembly contains a greater percentage of longer transcripts than previous assemblies. **b** Overlapping and distinct Nr database hits found in predicted protein-coding sequences in previous and current assemblies. The current assembly defines a greater number of new and distinct hits than previous assemblies

Comparison of the amino acid sequence length across the three sets of predicted protein coding sequences (Fig. 2a) found that the mean (ESTs: 196 aa; de novo: 470 aa; current: 569 aa), median (ESTs: 209 aa; de novo: 353 aa; current: 415 aa), and maximum (ESTs: 313; de novo: 8195 aa; current: 13,109 aa) sequence lengths in the current assembly were all higher than those in the ESTs library and de novo assembly. Comparison of unique Nr hits in the three sets of predicted protein-coding sequences showed that 7198 Nr hits were found in all three, with 3119, 1338, and 316 hits found only in the current assembly, de novo assembly, and ESTs library, respectively (Fig. 2b). Taken together, these findings indicate that the current combined genome-guided and de novo assembly improved the coverage and sequence length of the *L. stagnalis* CNS protein-coding transcriptome.

As shown in Fig. 2b, each of the three transcriptome libraries captured sequences that were absent from the other two. Therefore, we combined the three sets of sequences (31,520 sequences in total) to create the most comprehensive predicted protein-coding sequences library to date. These sequences were further clustered into a collection of 16,447 non-redundant predicted protein-coding sequences. BUSCO analysis showed that this set contains 96.2% of 978 single-copy orthologs present in > 90% of all eukaryotic species, higher than all three libraries when analyzed individually (Table 2). We next examined this comprehensive set of sequences to characterize gene expression in the *L. stagnalis* CNS.

### Comparisons of CNS transcriptomes of key neuroscience model organisms

**Fig. 3:**
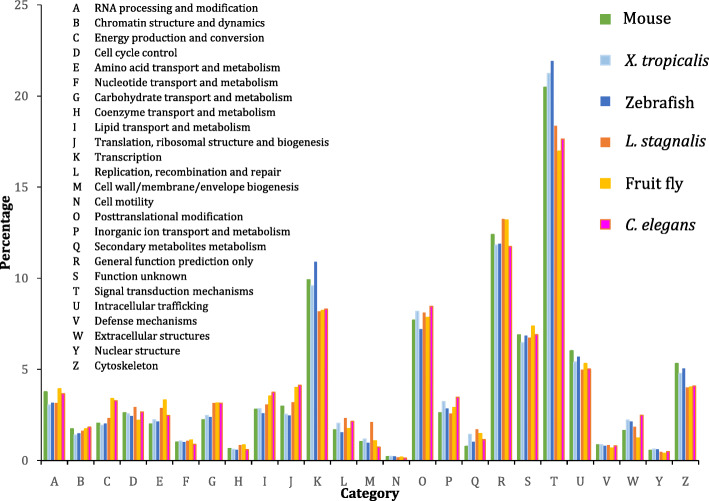
Comparison of KOG annotations of protein-coding transcripts expressed in the CNS of key vertebrate and invertebrate neuroscience model organisms (E-value <1E-5). Markedly, compared to invertebrates, vertebrate model organisms display increased percentage of transcripts involved in transcription, intracellular trafficking, and cytoskeleton. Meanwhile, compared to vertebrates, invertebrate model organisms display increased percentage of transcripts involved in energy production and conversion, amino acid transport and metabolism, carbohydrate transport and metabolism, and lipid transport and metabolism

This improved *L. stagnalis* CNS transcriptome provides an unprecedented opportunity to carry out comparative studies of CNS gene expression in a number of commonly used vertebrate and invertebrate neuroscience model organisms, so as to gain insights into commonalities and differences that may inform future studies. To this end, we analyzed publicly deposited RNA-seq libraries to characterize gene expression in the CNS of adult *Mus musculus* (mouse) [53], *Xenopus tropicalis* [53], *Danio rerio* (zebrafish) [54], *Drosophila melanogaster* (fruit fly) (BioProject: PRJNA320764), and *Caenorhabditis elegans* [55] (Table S1). Due to differing levels of non-coding RNA annotation for each species, we have focused our analyses on protein-coding transcripts. Annotation of the top 20 expressed transcripts in each of these five species (Tables S7, S8, S9, S10 and S11), in the same manner as done for *L. stagnalis* (Table 1), found a predominance of transcripts involved in energy production, protein synthesis, and signaling, in both vertebrate and invertebrate species. To gain a complementary perspective, we characterized the functional categorization of CNS protein-coding transcripts amongst *L. stagnalis* and these five common model organisms (Fig. 3) using their KOG annotations. Interestingly, the general distribution was largely similar, with “Signal transduction mechanisms” being the most abundant category overall. Nevertheless, in all three of the vertebrate species examined, more transcripts were functionally classified as “Signal transduction mechanisms”, “Transcription”, and “Cytoskeleton” than in the three invertebrate species. Conversely, in the three invertebrate species, more transcripts were identified to be functionally involved in “Carbohydrate transport and metabolism”, “Lipid transport and metabolism”, and “Translation, ribosomal structure and biosynthesis” than those in the three vertebrate species.

**Fig. 4:**
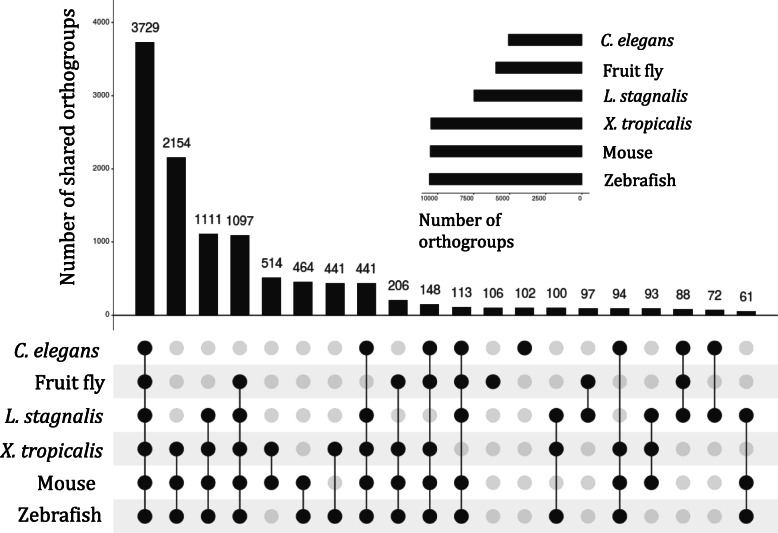
Membership of species in the orthogroups identified amongst protein-coding transcripts expressed in the CNS of *M. musculus*, *X. tropicalis,* *D. rerio*, *L. stagnalis*, *D. melanogaster*, and *C. elegans*. For clarity, only the 20 largest sets of shared orthogroups are shown. Dark and grey circles represent presence and absence, respectively, of the given species in each set of shared orthogroups. The number of orthogroups that each species is present in is shown in the inset bar graph. All species share the greatest number of orthogroups, followed by all vertebrates. Strikingly, of all three invertebrate species, *L. stagnalis* shares the greatest number of orthogroups with the three vertebrate species (1111)

**Fig. 5:**
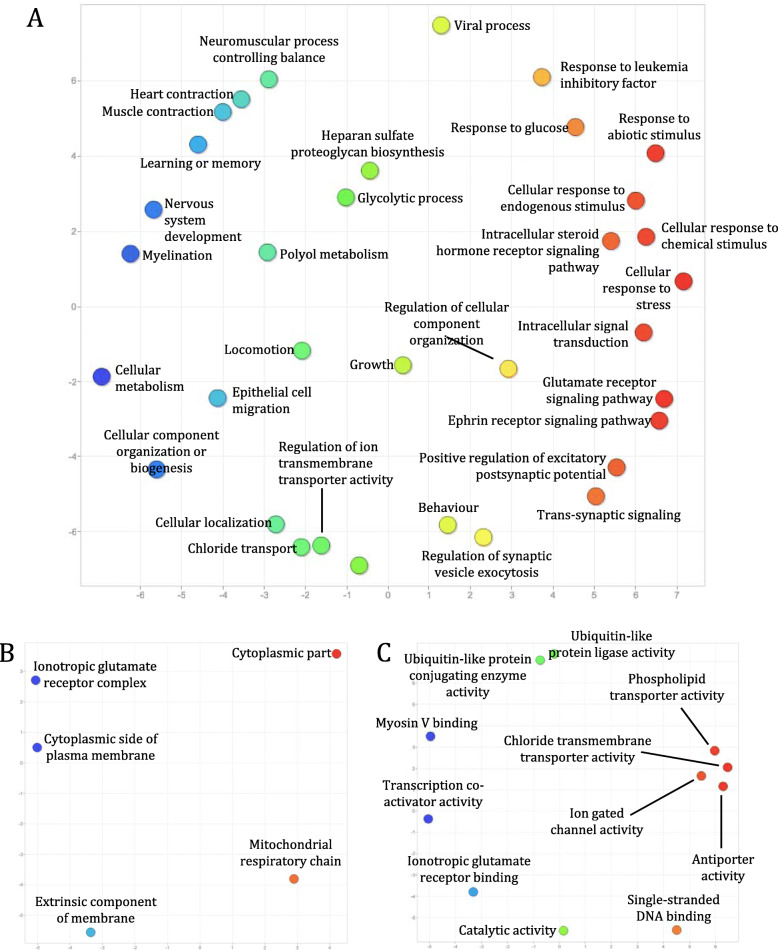
Enriched GO terms of genes encoded by transcripts in the 3729 orthogroups shared amongst *M. musculus*, *X. tropicalis,* *D. rerio*, *L. stagnalis*, *D. melanogaster*, and *C. elegans*. Using the REVIGO tool, GO terms in the “Biological process” (**a**), “Cellular components” (**b**) and “Molecular function” (**c**) classes were clustered in two-dimensional space via the SimRel semantic similarity measure to summarize their relationship to each other and coloured according to their positions on the semantic x-axis. Consequently, related GO terms are closer on the plot and more similar in colour. The arbitrary semantic space units on either axis have no intrinsic meaning. Orthogroups common amongst all six organisms commonly involved **a** intracellular signaling pathways (red/orange), **b** ionotropic glutamate receptor signaling (blue), and **c** ion transport (red/orange), among others

**Fig. 6:**
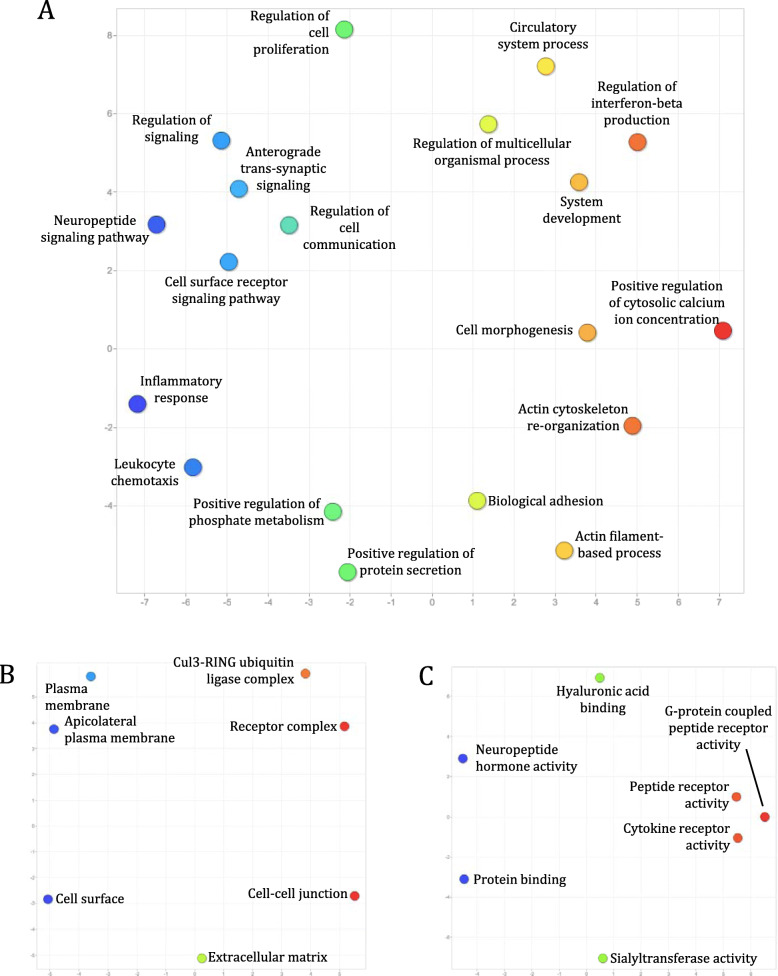
Enriched GO terms of genes encoded by transcripts in the 2154 orthogroups shared amongst only *M. musculus*, *X. tropicalis,* and *D. rerio*. GO terms in the “Biological process” (**a**), “Cellular components” (**b**) and “Molecular function” (**c**) classes are clustered and coloured in the same manner as described in Fig. 5. Orthogroups unique to these vertebrates commonly involved **a** intercellular communication (blue/green), **b** plasma membrane structure (blue), and **c** G-protein coupled, peptide, and cytokine receptor activity (red/orange), among others

**Fig. 7:**
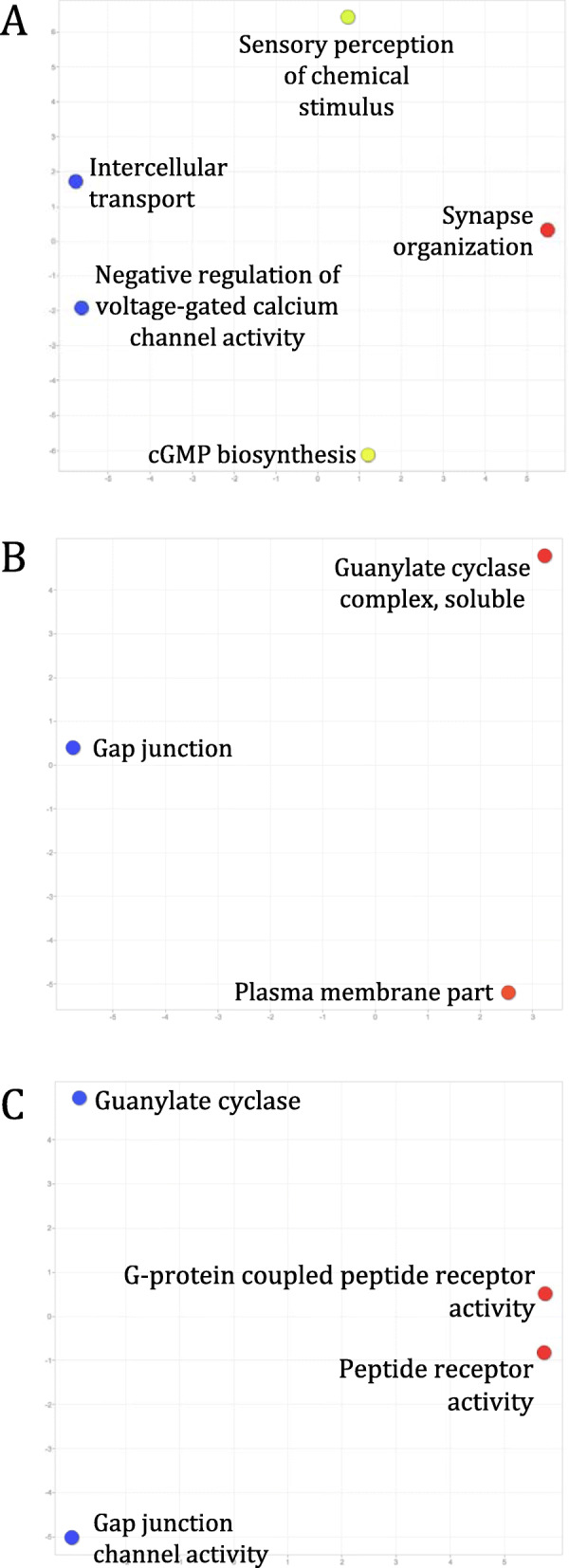
Enriched GO terms of genes encoded by transcripts in the 88 orthogroups shared amongst only *L. stagnalis*, *D. melanogaster*, and *C. elegans*. GO terms in the “Biological process” (**a**), “Cellular components” (**b**) and “Molecular function” (**c**) classes are clustered and coloured in the same manner as described in Fig. 5. Orthogroups unique to these invertebrates commonly involved **a** voltage-gated calcium channel activity regulation (blue), **b** plasma membrane structure (red), and **c** G-protein coupled peptide and peptide receptor activity, among others

We further clustered CNS protein-coding transcripts in the six species into orthologous groups to identify core CNS functions that are mediated by orthologous genes across distantly related species. In total, we identified 12,022 orthogroups, with 3729 orthogroups containing genes from all of the six species examined. Interestingly, *L. stagnalis* shares more orthogroups with the three vertebrate species than with *C. elegans* or fruit fly (Fig. 4). Functional enrichment of mouse genes (as they have the best annotation quality) in orthogroups shared amongst all species identified a variety of key CNS functions, including “Myelination” (GO:0042552), “Glutamate receptor signaling pathway” (GO:0007215), “Mitochondrial respiratory chain” (GO:0005746), and “ionotropic glutamate receptor binding” (GO:0035255) (Fig. 5; Table S12). These findings were complemented by Reactome (Table S13) and KEGG (Table S14) pathway analyses of the same set of mouse genes, which further identified known core CNS functions including “Axon guidance” (REAC:R-MMU-422475), “Cellular response to hypoxia” (REAC: R-MMU-2262749), “cAMP signaling pathway” (KEGG:04024) and “Insulin signaling pathway” (KEGG:04910). Furthermore, we identified 222 transcription factors whose binding motifs were enriched in the promoter region of this set of mouse genes (Table S15), identifying both well-characterized neuronal transcription factors such as CREB1 and AP2, but also novel proteins that may provide targets of future studies. We also performed similar enrichment analyses of genes in the 2154 vertebrate-only and 88 invertebrate-only orthogroups, in order to gain insights into differential functional specializations of the vertebrate and invertebrate CNS. Enrichment of mouse genes in the vertebrate-only orthogroups showed a constellation of GO (Fig. 6; Table S16), Reactome (Table S17) and KEGG (Table S18) terms related to immune/inflammatory processes, including “Leukocyte chemotaxis” (GO:0030595), “Regulation of interferon-beta production” (GO:0032648), “Complement and coagulation cascades” (KEGG:04610), and “Receptor-type tyrosine-protein phosphatases” (REAC:R-MMU-388844). Enrichment of fruit fly genes in the invertebrate-only orthogroups found terms relating to response to chemical stimuli, such as “Sensory perception of chemical stimulus” (GO:0007606), “Peptide receptor activity” (GO:0001653), and “G-protein coupled peptide receptor activity” (GO:0008528) (Fig. 7; Table S19). No enriched Reactome or KEGG pathways were found for this group of genes.

### Identification of ion channels and ionotropic receptors expressed in the *L. stagnalis* CNS and interspecies comparison

**Fig. 8:**
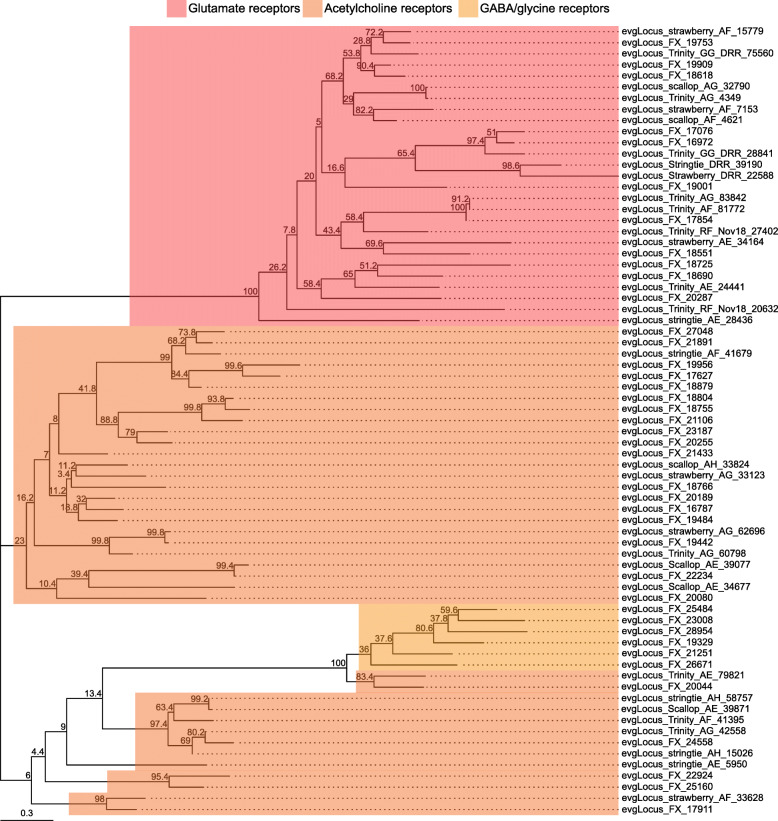
Ionotropic neurotransmitter receptor families identified in the *L. stagnalis* CNS transcriptome assembly. The tree with the highest log likelihood (− 7343.03) is shown. The percentage of trees in which the associated taxa clustered together (bootstrap values) is shown next to the branches. The tree is rooted at the mid-point and is drawn to scale, with branch lengths measured in the number of substitutions per site (see scale in bottom left). This analysis involved 33 amino acid sequences. There were a total of 133 positions in the final dataset. Evolutionary analyses were conducted in MEGA X (ver. 10.1.8) and tree visualization and annotation was conducted using the ggtree package (ver. 2.0.4) for R (ver. 3.6.3). This *L. stagnalis* transcriptome assembly identifies a wide diversity of putative acetylcholine, GABA/glycine, and glutamate receptors. The accession numbers and BLAST information of transcripts represented in this tree are provided in Tables S20, S21 and S22

**Fig. 9:**
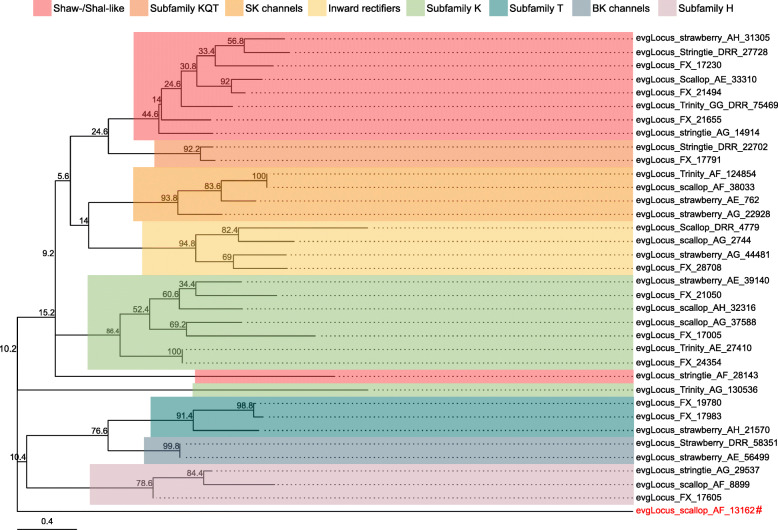
K^+^ channel subtypes and families identified in the *L. stagnalis* CNS transcriptome assembly. The tree was generated as described in Fig. 8, where the analysis involved 36 amino acid sequences and yielded a total of 75 positions in the final dataset, resulting in a tree with highest log likelihood of − 4511.88. The accession numbers and BLAST information of transcripts represented in this tree are provided in Table S23. A wide diversity of putative *L. stagnalis* K^+^ channel subtypes and families are identified, including a potentially novel family of voltage-gated K^+^ channels (denoted by red label, #)

**Fig. 10:**
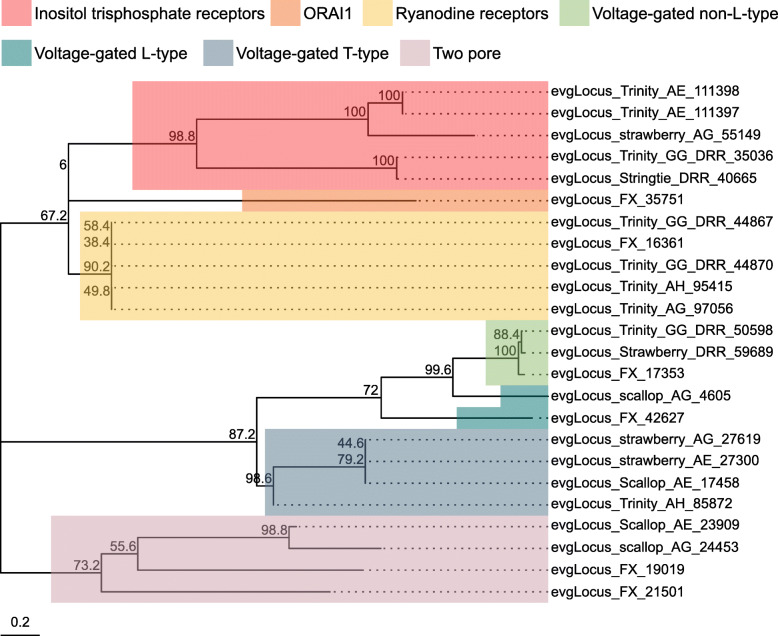
Ca^2+^ channel subtypes and families identified in the *L. stagnalis* CNS transcriptome assembly. The tree was generated as described in Fig. 8, where the analysis involved 24 amino acid sequences and yielded a total of 4018 positions in the final dataset, resulting in a tree with highest log likelihood of − 47,232.52. The accession numbers and BLAST information of transcripts represented in this tree are provided in Table S24. A diverse set of putative *L. stagnalis* Ca^2+^ channel subtypes and families are identified, including subtypes not yet cloned from *L. stagnalis*, such as Orai-2

**Fig. 11:**
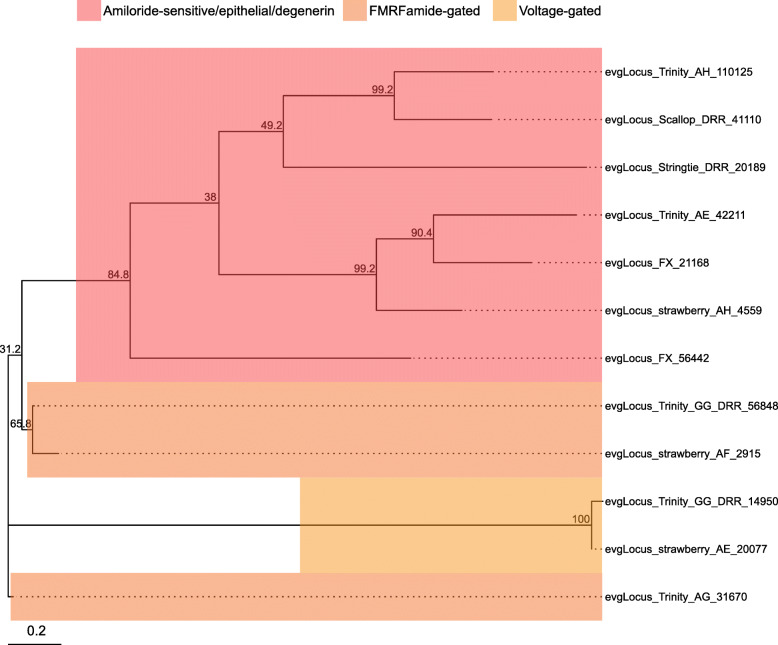
Na^+^ channel subtypes and families identified in the *L. stagnalis* CNS transcriptome assembly. The tree was generated as described in Fig. 8, where the analysis involved 12 amino acid sequences and yielded a total of 345 positions in the final dataset, resulting in a tree with highest log likelihood of − 6272.64. The accession numbers and BLAST information of transcripts represented in this tree are provided in Table S25. A variety of putative *L. stagnalis* Na^+^ channel subtypes and families are identified

**Fig. 12:**
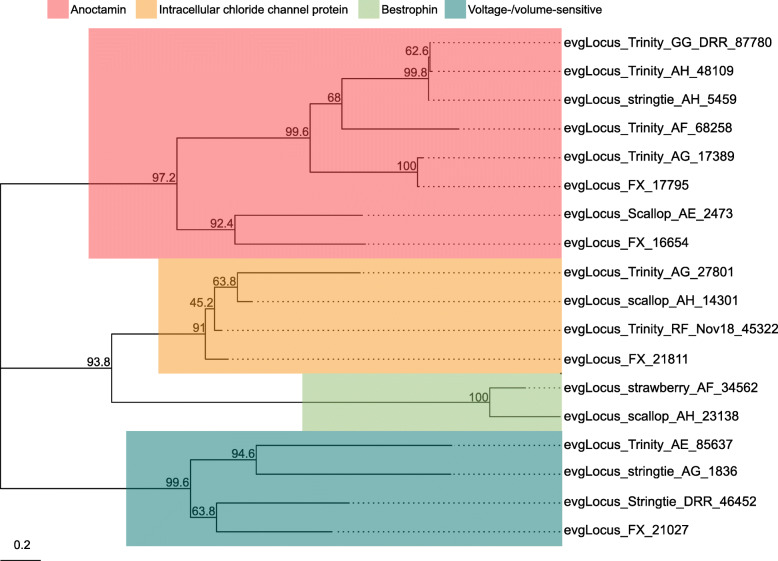
Cl^−^ channel subtypes and families identified in the *L. stagnalis* CNS transcriptome assembly. The tree was generated as described in Fig. 8, where the analysis involved 18 amino acid sequences and yielded a total of 1233 positions in the final dataset, resulting in a tree with highest log likelihood of − 22,161.37. The accession numbers and BLAST information of transcripts represented in this tree are provided in Table S26. A variety of putative *L. stagnalis* Cl^−^ channel subtypes and families are identified, of which anoctamin, bestrophin, and intracellular chloride channel protein have not yet been cloned from *L. stagnalis*

**Fig. 13:**
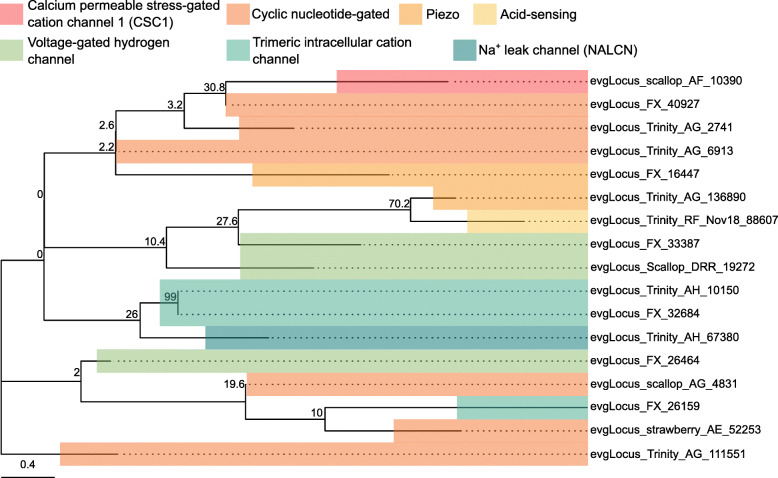
Cation channel subtypes and families identified in the *L. stagnalis* CNS transcriptome assembly. The tree was generated as described in Fig. 8, where the analysis involved 17 amino acid sequences and yielded a total of 8 positions in the final dataset, resulting in a tree with highest log likelihood of − 339.65. The accession numbers and BLAST information of transcripts represented in this tree are provided in Table S27. The majority of these diverse cation channels have not been cloned from *L. stagnalis*

**Fig. 14:**
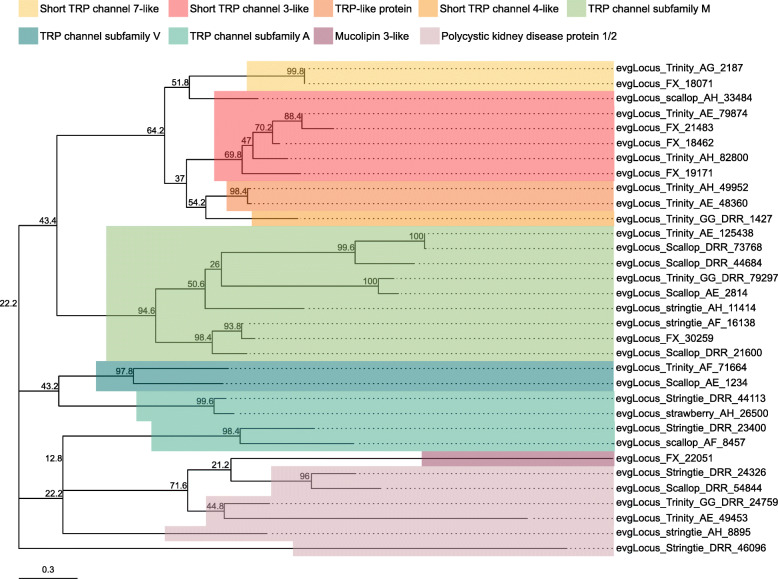
Transient receptor potential (TRP) channel subtypes and families identified in the *L. stagnalis* CNS transcriptome assembly. The tree was generated as described in Fig. 8, where the analysis involved 33 amino acid sequences and yielded a total of 133 positions in the final dataset, resulting in a tree with highest log likelihood of − 7343.03. The accession numbers and BLAST information of transcripts represented in this tree are provided in Table S28. This suggests a vertebrate-like diversity of TRP channels expressed by *L. stagnalis*

Ion channels and ionotropic neurotransmitter receptors play critical roles in regulating neuronal excitability and synaptic transmission in the CNS. As the simple and well-characterized *L. stagnalis* CNS is widely employed to study the fundamental mechanisms of neuronal function and circuit function [4, 56–58], we next sought to identify ion channels and ionotropic receptors expressed in the *L. stagnalis* CNS. We identified 211 protein-coding transcripts that contained both 1) ion channel or ionotropic receptor protein domains and 2) transmembrane helices to characterize the complement of ion channels and ionotropic receptors expressed in the *L. stagnalis* CNS. A proportion of sequences in this set were substantially shorter or longer than their best matched sequence in the Nr database. However, for the sake of completeness, we have included all in our analysis as some may be informative as splice isoforms. Sequence alignment-based phylogenetic analysis of these transcripts showed that they encode a wide range of ionotropic neurotransmitter receptors (Fig. 8; Tables S20, S21 and S22), K^+^ (Fig. 9; Table S23), Ca^2+^ (Fig. 10; Table S24), Na^+^ (Fig. 11; Table S25), Cl^−^ (Fig. 12; Table S26), cation (Fig. 13; Table S27), and transient receptor potential (TRP) (Fig. 14; Table S28) channels. Only 31 of the 211 putative ion channel/ionotropic receptor transcripts were annotated as a *L. stagnalis* sequence in the Nr database, indicating that majority of the sequences were identified here in this species for the first time.

**Fig. 15:**
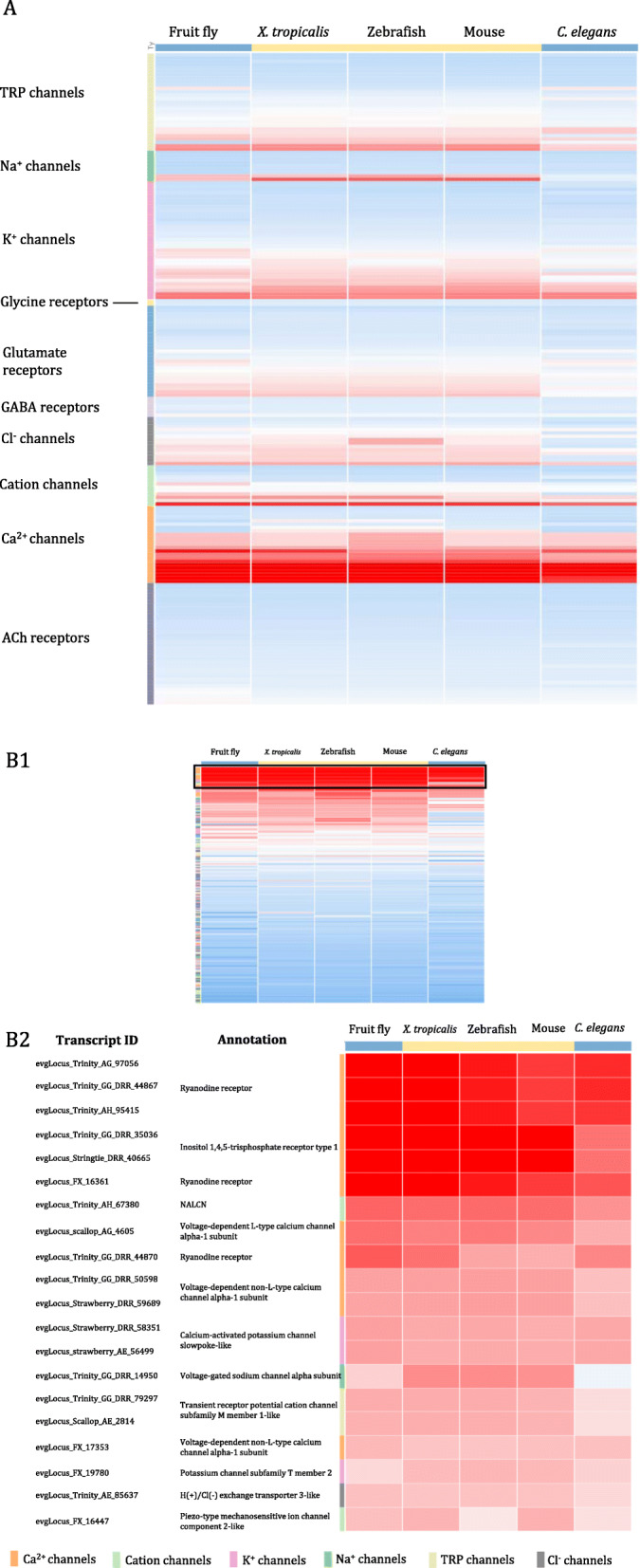
Heat maps of protein sequence similarity as measured by BLASTP bitscore between putative *L. stagnalis* ion channel/ionotropic receptor transcripts and homologs in mouse, *X. tropicalis,* zebrafish, fruit fly and *C. elegans*. Each bitscore is standardized by the average and standard deviation of all bitscores in the matrix. Each row corresponds to a single *L. stagnalis* transcript and each column to a species, such that each cell represents a standardized bitscore. Colour of each cell is proportional to the bitscore, where a darker colour indicates higher bitscore and consequently sequence similarity. **A**, Transcripts are sorted by the ion channel/ionotropic receptor class and species are sorted in descending order by the absolute sum of bitscores of its homologs, i.e. species with the highest bitscores across all transcripts is on the left of the heatmap. Transcripts encoding Ca^2+^ and cation channels appear to share the greatest sequence similarity across all six species. **B1** Transcripts are sorted in descending order by the absolute sum of bitscores, i.e. transcripts with the highest sequence similarities across species are at the top of the heatmap. **B2** The top 20 transcripts thus ranked are labeled by transcript ID and annotation. The colour scale is adjusted as compared to in **B1** to increase contrast between cells. Interactive Clustergrammer heat map can be accessed at http://amp.pharm.mssm.edu/clustergrammer/viz/5b6ba1ca7226c37ceda3505b/LS_channel_comparisons.txt. The majority of these top 20 transcripts encode calcium channels

We further examined the degrees of protein sequence similarity between ion channel/ionotropic receptor transcripts identified in *L. stagnalis* and those in these five model organisms through the BLASTp program and resulting bitscores of BLASTp hits. We plotted the standardized bitscore of each *L. stagnalis* ion channel/receptor against its top BLASTp hit in each species in a Clustergrammer heatmap (link provided in the figure legend), where bitscores lower and higher than the average are shown in blue and red, respectively (Fig. 15). Each row corresponds to a putative *L. stagnalis* ion channel/ionotropic receptor transcript and each column is one of the five species examined. First, ranking the species (columns) by the absolute sum of standardized bitscores, such that the species with the highest degree of sequence similarity with *L. stagnalis* is on the left of the graph, we observed that putative *L. stagnalis* ion channel/ionotropic receptor transcripts showed the most sequence similarity with their homologs in fruit fly and *X. tropicalis,* and the least with mouse and *C. elegans* (Fig. 15A)*.* Then, by sorting the transcripts according to their class (ex. Ca^2+^ channels, K^+^ channels, glutamate receptors, etc.), we found that *L. stagnalis* transcripts encoding Ca^2+^ channels showed the highest degrees of sequence similarities with their homologs as compared to all other classes (Fig. 15A). Finally, to more closely examine the individual *L. stagnalis* transcripts that showed the highest sequence similarities with other species, we sorted the transcripts (rows) by their absolute bitscore sum and focused on the top 20 transcripts (Fig. 15B). Indeed, in this list we observed numerous putative Ca^2+^ channel transcripts, such as ryanodine receptors (RyR), inositol 1,4,5-trisphosphate receptors, and voltage-gated Ca^2+^ channels (both L- and non-L-type). However, there were also a diversity of cation, K^+^ and TRP channels in this list. Taken together, these findings provide not only a resource for investigating the molecular mechanisms of CNS neural transmission and neuronal excitability in *L. stagnalis,* but also a foundation for better understanding the conservation and evolution of these processes across species.

## Discussion

*L. stagnalis* possesses a simple and well-characterized CNS that has been widely employed to elucidate key mechanisms of neural function [2, 4, 59–63], response to environmental pollutants [9, 10, 13], and resistance to parasitic infection [64]. However, molecular characterization of the CNS has been impeded by the lack of a comprehensive transcriptome. Here, we carried out the first genome-guided assembly of the *L. stagnalis* CNS transcriptome and integrated it with existing sequence databases to create the most comprehensive to-date transcriptome resource.

We took a combination of approaches to improve upon the existing sequence information. Firstly, we performed strand-specific paired-end RNA-seq that produced 150 bp long reads in this study, as compared to the unstranded 100 bp single-end reads used in the previously published de novo assembly [50]. This allows for better unique mapping to the reference genome and splice junction detection, and thus more accurate reconstruction of transcripts [65]. Secondly, we leveraged the strengths of multiple assembly software packages and both genome-guided and de novo assembly methods, which have been shown to increase transcriptome completeness and limit sequence redundancy [66, 67]. We further added to the diversity of the final transcript set by including previously published ESTs [49] and de novo assembled transcripts [50]. Finally, we used a combination of ORF detection, expression-based filtering, protein domain identification and clustering methods to remove sequencing/assembly artefacts and fragmented/redundant sequences in this aggregated set, creating a collection of high-confidence predicted protein-coding transcripts (available on http://lymnaea.org). Indeed, we have shown that this transcript set exceeds existing resources in terms of sequence length, completeness, and diversity (Fig. 2, Table 2).

In this study, we functionally characterized the *L. stagnalis* CNS transcriptome and identified transcripts critical for electrical excitability and neurotransmission. First, we demonstrated that annotated or predicted neuropeptides predominate the list of top 20 most abundantly expressed transcripts, which is consistent with the importance of neuropeptide signaling in regulating a variety of physiological processes in *L. stagnalis*, including neuronal excitability [68], heartbeat [69], feeding [70], and reproduction [71, 72]. This group of transcripts is strikingly similar to most highly expressed transcripts observed in the CNS of two other mollusc species, *Tritonia diomedea* [73] and *Biomphalaria alexandrina* [74], which also include transcripts encoding soma ferritin, acetylcholine-binding protein, and numerous neuropeptides such as sodium-influx-stimulating peptide, molluscan insulin-related peptide 3 and cardioexcitatory peptide. Then, we characterized the functional distribution of protein-coding transcripts expressed in the *L. stagnalis* CNS through KOG annotations (Fig. 3), showing that they are predominantly involved in mediating signaling transduction, general function and transcription processes. Finally, for the first time, we systematically identified transcripts that putatively encode 43 subtypes of Na^+^, K^+^, Ca^2+^, Cl^−^ and cation ion channels and ionotropic neurotransmitter receptors (Figs. 8, 9, 10, 11, 12, 13 and 14, Tables S15, S16, S17, S18, S19, S20, S21, S22 and S23), greatly expanding our understanding of the molecular determinants of neural excitability and transmission in the *L. stagnalis* CNS. As the *L. stagnalis* CNS has been widely employed to study ion channels and/or ionotropic neurotransmitter receptors [38, 75–77], we examined the protein sequence similarity of each identified *L. stagnalis* channel/receptor transcripts with its homolog in *M. musculus*, *X. tropicalis, D. rerio*, *D. melanogaster*, and *C. elegans.*

Consistent with the importance of intracellular Ca^2+^ dynamics and homeostasis for neuronal function [78], we found that transcripts encoding the intracellular calcium release channels, ryanodine receptors and inositol triphosphate receptors, and voltage-gated Ca^2+^ channels in *L. stagnalis* show the highest degrees of sequence similarity with their homologs in the other five species (Fig. 15). The non-selective Na^+^-leak channel NALCN also exhibited a high degree of sequence conservation between *L. stagnalis* and the other five species, which is consistent with the necessary role of NALCN in regulating basal membrane potential in *L. stagnalis* [62, 75], *M. musculus* [79], *D. melanogaster* [80] and *C. elegans* neurons [81]. Interestingly, a member of the TRP channel family, transient receptor potential melastatin 1 (TRPM1), also showed a moderate degree of sequence conservation between species. Lower bitscores are consistent with the fact that invertebrate TRPM channels may be divided into broader α- and β-subfamilies, unlike vertebrates, which express TRPM1–8 [82, 83]. This relative conservation may point to a conserved function of TRPM1 in phototransduction across organisms, as has been identified in *M. musculus* and *D. melanogaster* [84], suggesting future investigation may be warranted in studying the role of TRPM1-like proteins in *L. stagnalis* phototransduction. Meanwhile, the acetylcholine receptor family of ligand-gated ion channels in *L. stagnalis* showed a low degree of sequence similarity with other species. This corresponds well with known expansions in acetylcholine receptor sequences, particularly nicotinic acetylcholine receptors, in molluscs as compared to vertebrate species [85], and consistent with previous studies on *L. stagnalis* acetylcholine receptors [86]. Taken together, this study provides the resources for future genetic and bioinformatic studies of various ion channels, thus opening up exciting new venues for exploring fundamental and novel mechanisms of neural function in *L. stagnalis.*

As *L. stagnalis* is a key model organism used to examine both conserved and novel mechanisms of neurobiology, it is of immense interest to compare and contrast its CNS gene expression with that of other common neuroscience model organisms, in order to better understand the extent to which CNS functions are conserved and consequently how generalizable experimental findings are across species. Here, we compared *L. stagnalis* CNS gene expression with that of *M. musculus*, *X. tropicalis*, *D. rerio, D. melanogaster*, and *C. elegans* using several approaches. A comparison of the top 20 most abundantly expressed protein-coding transcripts in these five species (Table 1, Tables S7, S8, S9, S10 and S11) with those in *L. stagnalis* found several common functional categories critical for CNS function, including energy production, signaling and protein synthesis. Surprisingly, no clear distinctions in terms of prevalent functions were observed between the three vertebrate and three invertebrate species. Comparison of KOG annotations of all the expressed protein-coding transcripts in each species provides a complementary perspective (Fig. 3). While the distribution of transcripts across functional categories was largely similar across the six species, vertebrate species were shown to have more transcripts involved in signal transduction, gene expression and cytoskeleton dynamics, whereas in invertebrates, more transcripts were shown to be related to metabolism and protein synthesis. In the case of cytoskeleton dynamics, this is consistent with known differences in multidirectional polarity and absence of dendritic spines in invertebrate neurons as compared to their vertebrate counterparts [87]. On the other hand, reasons for enrichment of transcripts related to metabolism and protein synthesis in invertebrates are less clear, especially given that metabolic regulation is generally less complex in invertebrates than vertebrates [88]. However, short neuropeptide F signaling, which is not present in vertebrates and plays a role in regulating metabolism and energy homeostasis in invertebrates [89], may account for this difference in metabolism KOG annotations. Subsequently, differences in metabolic pathways between vertebrate and invertebrate CNS may be an interesting avenue of future study.

Finally, we clustered all protein-coding transcripts expressed in the CNS of these six species into orthogroups, each of which was predicted based on sequence similarities to be descended from a common gene in the last common ancestor of these six species. As orthologous genes have been shown to exhibit similar functions [90, 91] and expression patterns [92] across species, such analyses may provide insights into conserved CNS functions across divergent species. Indeed, functional enrichment of genes encoded by transcripts in orthogroups shared amongst all six species identified processes fundamental to CNS functioning, such as glutamate receptor signaling, ion transport, and mitochondrial aerobic respiration (Fig. 5). Conversely, analyses of orthogroups shared amongst only the vertebrate (Fig. 6) or invertebrate (Fig. 7) species examined showed that functions related to the immune/inflammatory response, actin dynamics and G protein-coupled receptor (GPCR) signaling were likely less conserved between the two groups. This is expected, as invertebrates lack acquired immune responses and thus the genes associated with acquired immunity, relying entirely on innate immunity to defend against infection [93, 94]. Similarly, most invertebrates lack dendritic spines, plasticity of which relies on actin remodeling [95], and differential conservation of actin dynamics between vertebrates and invertebrates identified in the current study is consistent with this. Finally, differences in enrichment of genes related to GPCR signaling is expected due to differences in the ensemble of GPCRs expressed in invertebrates compared to vertebrates. For instance, *C. elegans* expresses nematode chemosensory (NCHM) GPCRs, which are not present in vertebrates [96]. Additionally, some invertebrates lack various Gα proteins, including Gαi/o and Gαq subtypes [97]. A full characterization of GPCRs in *L. stagnalis* may be thus of interest for future study.

## Conclusion

We have shown that *L. stagnalis* had more orthogroups in common with the three vertebrate species (*M. musculus*, *D. rerio*, and *X. tropicalis*) than *D. melanogaster* or *C. elegans,* suggesting that it may be an advantageous system in which to leverage the simplicity of an invertebrate CNS but also maintain more conserved molecular mechanisms with higher organisms. To the best of our knowledge, there have been few systematic comparisons of CNS physiology in commonly used vertebrate and invertebrate model organisms. Such comparisons can provide insights into the evolutionary history of various cellular processes and protein families. Our findings in this study provide a starting point for better understanding of the functional commonalities and specializations of the vertebrate and invertebrate CNS, which is critically needed to inform future studies in terms of the suitability of the chosen model and generalizability of future findings.

## Methods

### RNA-seq and pre-processing of reads

The central ganglia ring from four adult *L. stagnalis* were dissected according to established protocols [49, 63, 98, 99] and total RNA was extracted using the Trizol method as previously described [7]. RNA quality was evaluated by capillary electrophoresis migration on an Agilent Bioanalyzer, using the Agilent RNA Pico 6000 LabChip kit. The RNA integrity number (RIN) score was not able to be calculated due to the lack of 28S RNA-associated peak on electropherograms of *L. stagnalis* RNA. This phenomenon occurs due to a thermolabile hidden break in the molecule, as described in various invertebrates, which leads to its cleavage into two sub-parts of size similar to that of 18S RNA [100]. For this reason, RNA quality was evaluated using the global shape of migration profiles. One 150 bp paired-end RNA-seq library was prepared from 500 μg of total RNA from each central ring ganglia sample, using the TruSeq Stranded mRNA kit (Illumina, San Diego, CA, USA). Each library was sequenced using 151-bp paired-end reads chemistry on a HiSeq 4000 Illumina sequencer, where 20 to 22 million reads were generated per library and all 4 libraries were sequenced on a single lane. The raw data were filtered to remove any clusters that had too high intensity corresponding to bases other than the called base. By default, the purity of the signal from each cluster was examined over the first 25 cycles and calculated as Chastity = Highest_Intensity / (Highest_Intensity + Next_Highest_Intensity) for each cycle. The default filtering implemented at the base calling stage allows at most one cycle that is less than the Chastity threshold (0.6). The reads were further processed by trimming, using the fastx_clean script from the FASTX toolkit [101]: 1) adapters and primers on the whole read, 2) low quality nucleotides from both ends (where quality value was lower than 20), and 3) sequences between the second unknown nucleotide (N) and the end of the read. Finally, reads that either 1) were shorter than 30 nucleotides, 2) originated from the low-concentration spike-in library of Illumina PhiX Control or 3) corresponded to ribosomal RNA, as filtered by SortMeRNA v 1.0 [102] were removed.

### Transcriptome assembly and annotation of predicted protein-coding transcripts

Workflow for assembling and identifying protein-coding transcripts in the *L. stagnalis* CNS transcriptome is summarized in Fig. 1. Read quality was visualized using FastQC [103], and then analyzed using rCorrector [104], which employs a k-mer based method to correct random sequencing errors in Illumina RNA-seq reads. Reads were mapped to the genome assembly using STAR [105] and assembled using Stringtie [106], Scallop [107], Strawberry [108], and Trinity [109] genome-guided assembly programs, as well as Trinity de novo assembly program. This resulted in four genome-guided assemblies per sample (16), one de novo assembly per sample (4). Reads that were flagged as erroneous but unfixable were removed using a custom script (https://github.com/harvardinformatics/TranscriptomeAssemblyTools/blob/master/FilterUncorrectabledPEfastq.py), and processed and assembled in the same manner, except using a modified custom script to remove unfixable single-end reads after rCorrector analysis. This resulted in five additional assemblies: four genome-guided assemblies and one de novo assembly of unmapped reads. These 22 assemblies (including four per sample from each assembly program, one de novo unmapped assembly, and the above additional assemblies) were merged and processed through the EvidentialGene tr2aacds pipeline [110] to identify a set of non-redundant potential protein-coding transcripts, as previously described [111, 112]. This pipeline removes perfect duplicate sequences, identifies and clusters highly similar sequences through a “all-vs-all BLAST” (98% similarity by default) process, and identifies potential open reading frames (ORFs) in each transcript. The final set of predicted ORF-containing transcripts (> 100 bp) were sorted into three sets: “okay” for primary transcripts, “okalt” for alternative transcripts and “drops” for redundant, fragmented or too short transcripts. Transcripts containing complete or 3′-partial open ORFs were extracted from the primary and alternate sets for downstream analyses. Expression levels of the transcripts were assessed using Salmon [113] to allow for expression-based filtering of potential transcript artifacts (TPM > 0 in all five RNA-seq libraries). The resultant transcripts were searched against the Pfam database and analyzed using the signaling peptide identification programs SignalP and Phobius to assess their potential as protein-coding transcripts. All predicted protein coding sequences were annotated against the Swissprot, Nr, KOG databases and the *L. stagnalis* predicted gene set (E-value cut-off of 1E-5). BUSCO analysis [114] was performed to assess the completeness of this set of transcripts (E-value cut-off of 1E-5). The Salmon software was used to calculate the expression level of the predicted coding transcripts in each of the four paired-end reads libraries. The transcripts were ranked by their expression level in each of the four libraries sequenced for this study, and the median rank for each transcript was calculated across all four libraries to identify the top 20 expressed transcripts.

### Comparison with previously published *L. stagnalis* CNS transcriptome libraries and aggregation of protein-coding sequence library

For comparison with the current assembly, the previously published EST library [49] and de novo assembled transcripts [50] from the *L. stagnalis* CNS were obtained from the European Nucleotide Archive and processed in the same manner as described above to predict protein-coding sequences. The amino acid sequences were annotated against Nr database using BLASTP (E-value cut-off of 1E-5). Finally, the amino acid sequences of predicted protein-coding sequences from these two libraries and the current assembly were clustered and deduplicated (100% similarity cut-off) separately using CD-HIT [115]. The resultant set of sequences was analyzed by BUSCO to assess completeness (E-value cut-off of 1E-5).

### Analyses of published CNS RNA-seq libraries from representative model organisms of animals

Published RNA-seq libraries of *M. musculus* [53], *D. rerio* [54], *X. tropicalis* [53], *D. melanogaster* (PRJNA320764), and *C. elegans* [55] CNS were obtained from the NCBI Short Reads Archive (Table S1). Where possible, equal numbers of sequencing libraries from both male and female animals were analyzed. Quality of reads was visualized using FastQC, and erroneous bases were corrected and filtered in the same manner as described above. The fastp program [116] was then employed to remove adapters and trim bases with phred score < 5, as previously recommended [117]. The software Salmon [113] was used to map reads to reference transcripts (both cDNAs and non-coding RNAs) for each species. Protein sequences of transcripts with TPM > 0 in all libraries examined for the species were collected for all species examined and clustered using Orthofinder [118] to identify orthogroups. Membership of each species in orthogroups were visualized using UpSetR [119]. Gene IDs of protein-coding transcripts with TPM > 0 in all libraries of each species were retrieved from Biomart via the biomaRt R package [120] for functional enrichments using g:Profiler R package [121], where IEA were excluded and statistical significance was calculated using the gSC method (p-adjusted< 0.05 cut-off). Clustering of Gene Ontology (GO) terms was visualized using REViGO [122].

### Identification of transcripts encoding ion channels and ionotropic receptors and interspecies comparisons

Pfam IDs containing the word “channel” in their descriptions were obtained from Pfam (pfam.xfam.org) (Table S2). Predicted protein-coding transcripts containing at least one of this set of protein domains were then analyzed using Phobius and TMHMM, and only those predicted to contain transmembrane helices by both programs were retained. The putative ion channel/receptor sequences were annotated against the Nr database (E-value cut-off of 1E-5) and manually inspected to separate into ionotropic neurotransmitter receptor and Na^+^, K^+^, Ca^2+^, Cl^−^, and cation channel subfamilies. The sequences within each subfamily were aligned and clustered using T-Coffee [123], trimmed using trimAl [124], and phylogenetic trees were computed using the Maximum Likelihood method and JTT matrix-based model [125] in MEGA X [126]. Initial tree(s) for the heuristic search were obtained automatically by applying Neighbor-Join and BioNJ algorithms to a matrix of pairwise distances estimated using the JTT model, and then selecting the topology with superior log likelihood value. Phylogenetic trees were visualized using the ggtree package for R [127].*M. musculus*, *X. tropicalis, D. rerio*, *D. melanogaster*and *C. elegans* homologs of the putative *L. stagnalis* ion channel/receptor transcripts were identified via BLASTP (E-value cut-off of 1E-5). The heatmap of standardized BLAST bitscores was visualized using Clustergrammer [128].

## Supplementary Information

**Additional file 1: Table S1.** Published adult CNS RNA-seq libraries from selected model organisms used in this study. **Table S2.** Pfam IDs used to identify transcripts encoding ion channels and ionotropic receptors in the *L. stagnalis* CNS*.* **Table S3.** Summary of *L. stagnalis* CNS RNA-seq library metrics before and after reads correction and filtering. **Table S4.** Mapping statistics of the CNS RNA-seq libraries to *L. stagnalis* genome assembly. **Table S5.** Transcript assembly statistics for *L. stagnalis* CNS RNA-seq libraries. **Table S6.** Proportion of transcripts containing complete and fragmented ORFs as identified by the Evigene pipeline in the “okay” and “okalt” sequence sets. **Table S7.** Top 20 expressed transcripts in the adult mouse brain. **Table S8.** Top 20 expressed transcripts in the adult *X. tropicalis* brain. **Table S9.** Top 20 expressed transcripts in the adult zebrafish brain. **Table S10.** Top 20 expressed transcripts in the adult fruitfly brain. **Table S11.** Top 20 expressed transcripts in adult *C. elegans* neurons. **Table S12.** Enriched GO terms of mouse genes in orthogroups shared amongst vertebrate and invertebrate species. **Table S13.** Enriched Reactome pathways of mouse genes in orthogroups shared amongst all the species examined. **Table S14.** Enriched KEGG pathways of mouse genes in orthogroups shared amongst all the species examined. **Table S15.** Transcript factors whose binding motifs are enriched in the set of mouse genes in orthogroups shared amongst all the species examined. **Table S16.** Enriched GO terms of mouse genes in orthgroups shared amongst only the vertebrate species examined. **Table S17.** Enriched Reactome pathways of mouse genes in orthgroups shared amongst only the vertebrate species examined. **Table S18.** Enriched KEGG pathways of mouse genes in orthgroups shared amongst only the vertebrate species examined. **Table S19.** Enriched GO terms of fruit fly genes in orthgroups shared amongst only the invertebrate species examined.

**Additional file 2: Table S20.** Transcripts encoding ionotropic acetylcholine receptors in the *L. stagnalis* CNS. **Table S21.** Transcripts encoding ionotropic GABA or glycine receptors in the *L. stagnalis* CNS. **Table S22.** Transcripts encoding ionotropic glutamate receptors in the *L. stagnalis* CNS. **Table S23.** Transcripts encoding K+ channels in the *L. stagnalis* CNS. **Table S24.** Transcripts encoding Ca2+ channels in the *L. stagnalis* CNS. **Table S25.** Transcripts encoding Na + channels in the *L. stagnalis* CNS. **Table S26.** Transcripts encoding Cl- channels in the *L. stagnalis* CNS. **Table S27.** Transcripts encoding cation channels in the *L. stagnalis* CNS. **Table S28.** Transcripts encoding TRP channels in the *L. stagnalis* CNS.

## Data Availability

The raw sequence reads have been deposited in the Sequence Read Archive (SRA) under accession numbers ERX4742139-ERX4742142, and all associated project data are available under the study identifier PRJEB41522 at NCBI or EBI (as well as http://www.lymnaea.org/).

## Abbreviations

AA: Amino acid
BLAST: Basic local alignment search tool
CNS: Central nervous system
CPG: Central pattern generator
CRAC: Calcium release-activated current
EST: Expressed sequence tag
GABA: Gamma-aminobutyric acid
GO: Gene ontology
GPCR: G protein-coupled receptor
HCN: Hyperpolarization-activated cyclic nucleotide-gated
KEGG: Kyoto Encyclopedia of Genes and Genomes
KOG: Eukaryotic orthologous group
NALCN: Sodium (Na^+^) leak channel non-selective
ORF: Open reading frame
RIN: RNA integrity number
RNA: Ribonucleic acid
RyR: Ryanodine receptors
TPM: Transcripts per kilobase million
TRP: Transient receptor potential
