# Supplementary material for: Ion channel profiling of the Lymnaea stagnalis ganglia via transcriptome analysis
Source: BMC Genomics. 2021 Jan 6;22:18. doi: 10.1186/s12864-020-07287-2 (PMC7789530; doi:10.1186/s12864-020-07287-2)
Supplement: Supplementary file 1 — Additional file 1: Table S1. Published adult CNS RNA-seq libraries from selected model organisms used in this study. Table S2. Pfam IDs used to identify transcripts encoding ion channels and ionotropic receptors in the L. stagnalis CNS. Table S3. Summary of L. stagnalis CNS RNA-seq library metrics before and after reads correction and filtering. Table S4. Mapping statistics of the CNS RNA-seq libraries to L. stagnalis genome assembly. Table S5. Transcript assembly statistics for L. stagnalis CNS RNA-seq libraries. Table S6. Proportion of transcripts containing complete and fragmented ORFs as identified by the Evigene pipeline in the “okay” and “okalt” sequence sets. Table S7. Top 20 expressed transcripts in the adult mouse brain. Table S8. Top 20 expressed transcripts in the adult X. tropicalis brain. Table S9. Top 20 expressed transcripts in the adult zebrafish brain. Table S10. Top 20 expressed transcripts in the adult fruitfly brain. Table S11. Top 20 expressed transcripts in adult C. elegans neurons. Table S12. Enriched GO terms of mouse genes in orthogroups shared amongst vertebrate and invertebrate species. Table S13. Enriched Reactome pathways of mouse genes in orthogroups shared amongst all the species examined. Table S14. Enriched KEGG pathways of mouse genes in orthogroups shared amongst all the species examined. Table S15. Transcript factors whose binding motifs are enriched in the set of mouse genes in orthogroups shared amongst all the species examined. Table S16. Enriched GO terms of mouse genes in orthgroups shared amongst only the vertebrate species examined. Table S17. Enriched Reactome pathways of mouse genes in orthgroups shared amongst only the vertebrate species examined. Table S18. Enriched KEGG pathways of mouse genes in orthgroups shared amongst only the vertebrate species examined. Table S19. Enriched GO terms of fruit fly genes in orthgroups shared amongst only the invertebrate species examined. [file 12864_2020_7287_MOESM1_ESM.pdf]

Table S1. Published adult CNS RNA-seq libraries from selected model organisms used in this study.

	**Mouse**	***X. tropicalis***	**Zebrafish**	**Fruit fly**	***C. elegans***
**Reference**	Marin *et al.*, 2017	Marin *et al.*, 2017	Modzelewska *et al.*, 2016	Unpublished	Kaletsky *et al.*, 2017
**BioProject**	PRJNA381064	PRJNA381064	PRJNA319956	PRJNA320764	PRJNA400796
**Age**	9-weeks	4-6 month	4-6 weeks	3-days post-eclosion	Day 1 adult
**Libraries**	SRR5412186 (f)<br>SRR5412187 (f)<br>SRR5412188 (m)<br>SRR5412189 (m)	SRR5412261 (f)<br>SRR5412262 (f)<br>SRR5412263 (m)<br>SRR5412264 (m)	SRR3465546<br>SRR3465547<br>SRR3465548<br>SRR3465549	SRR3478195 (f)<br>SRR3478196 (f)<br>SRR3478197 (f)<br>SRR3478217 (m)<br>SRR3478218 (m)<br>SRR3478219 (m)	SRR7443976<br>SRR7443981<br>SRR7443983<br>SRR7443984
**Reference transcripts**	GRCm38 (M17)	JGI v9.1	GRCz11	BDGP6	WBcel235

* f: female
m: male

Table S2. Pfam IDs used to identify transcripts encoding ion channels and ionotropic receptors in the *L. stagnalis* CNS.

**ID**	**Name**	**Description**
PF00060	Lig_chan	Ligand-gated ion channel
PF00654	Voltage_CLC	Voltage gated chloride channel
PF00858	ASC	Amiloride-sensitive sodium channel
PF00924	MS_channel	Mechanosensitive ion channel
PF01007	IRK	Inward rectifier potassium channel
PF01062	Bestrophin	Bestrophin, RFP-TM, chloride channel
PF01618	MotA_ExbB	MotA/TolQ/ExbB proton channel family
PF01741	MscL	Large-conductance mechanosensitive channel, MscL
PF02060	ISK_Channel	Slow voltage-gated potassium channel
PF02702	KdpD	Osmosensitive K+ channel His kinase sensor domain
PF02714	RSN1_7TM	Calcium-dependent channel, 7TM region, putative phosphate
PF02931	Neur_chan_LBD	Neurotransmitter-gated ion-channel ligand binding domain
PF02932	Neur_chan_memb	Neurotransmitter-gated ion-channel transmembrane region
PF03185	CaKB	Calcium-activated potassium channel, beta subunit
PF03493	BK_channel_a	Calcium-activated BK potassium channel alpha subunit
PF03502	Channel_Tsx	Nucleoside-specific channel-forming protein, Tsx
PF03520	KCNQ_channel	KCNQ voltage-gated potassium channel
PF03521	Kv2channel	Kv2 voltage-gated K+ channel
PF03530	SK_channel	Calcium-activated SK potassium channel
PF03595	SLAC1	Voltage-dependent anion channel
PF04547	Anoctamin	Calcium-activated chloride channel
PF05197	TRIC	TRIC channel
PF05934	MCLC	Mid-1-related chloride channel (MCLC)
PF05978	UNC-93	Ion channel regulatory protein UNC-93
PF06011	TRP	Transient receptor potential (TRP) ion channel
PF06241	Castor_Poll_mid	Castor and Pollux, part of voltage-gated ion channel
PF07740	Toxin_12	Ion channel inhibitory toxin
PF07856	Orai-1	Mediator of CRAC channel activity
PF07885	Ion_trans_2	Ion channel
PF07936	Defensin_4	Potassium-channel blocking toxin
PF00520	Ion_trans	Ion transport protein
PF07941	K_channel_TID	Potassium channel Kv1.4 tandem inactivation domain
PF08016	PKD_channel	Polycystin cation channel
PF08344	TRP_2	Transient receptor ion channel II
PF08434	CLCA	Calcium-activated chloride channel N terminal
PF08466	IRK_N	Inward rectifier potassium channel N-terminal
PF08473	VGCC_alpha2	Neuronal voltage-dependent calcium channel alpha 2acd
PF08763	Ca_chan_IQ	Voltage gated calcium channel IQ domain
PF10613	Lig_chan-Glu_bd	Ligated ion channel L-glutamate- and glycine-binding site
PF11404	Potassium_chann	Potassium voltage-gated channel
PF11601	Shal-type	Shal-type voltage-gated potassium channels, N-terminal
PF11834	KHA	KHA, dimerisation domain of potassium ion channel
PF11933	Na_trans_cytopl	Cytoplasmic domain of voltage-gated Na+ ion channel
PF12052	VGCC_beta4Aa_N	Voltage gated calcium channel subunit beta domain 4Aa N terminal
PF12166	Piezo_RRas_bdg	Piezo non-specific cation channel, R-Ras-binding domain
PF12794	MscS_TM	Mechanosensitive ion channel inner membrane domain 1
PF12795	MscS_porin	Mechanosensitive ion channel porin domain
PF12929	Mid1	Stretch-activated Ca2+-permeable channel component
PF13677	MotB_plug	Membrane MotB of proton-channel complex MotA/MotB
PF13965	SID-1_RNA_chan	dsRNA-gated channel SID-1
PF14776	UNC-79	Cation-channel complex subunit UNC-79
PF14866	Toxin_38	Potassium channel toxin
PF15020	CATSPERD	Cation channel sperm-associated protein subunit delta

Table S2 (continued). Pfam IDs used to identify transcripts encoding ion channels and ionotropic receptors in the *L. stagnalis* CNS.

**ID**	**Name**	**Description**
PF15064	CATSPERG	Cation channel sperm-associated protein subunit gamma
PF15108	TMEM37	Voltage-dependent calcium channel gamma-like subunit protein family
PF15149	CATSPERB	Cation channel sperm-associated protein subunit beta protein family
PF15778	UNC80	Cation channel complex component UNC80
PF15803	zf-SCNM1	Zinc-finger of sodium channel modifier 1
PF15805	SCNM1_acidic	Acidic C-terminal region of sodium channel modifier 1 SCNM1
PF15968	RexB	Membrane-anchored ion channel, Abi component
PF16178	Anoct_dimer	Dimerisation domain of Ca+-activated chloride-channel, anoctamin
PF16526	CLZ	C-terminal leucine zipper domain of cyclic nucleotide-gated channels
PF16642	KCNQ2_u3	Unstructured region on Potassium channel subunit alpha KvLQT2
PF16799	VGPC1_C	C-terminal membrane-localisation domain of ion-channel, VCN1
PF16885	CAC1F_C	Voltage-gated calcium channel subunit alpha, C-term
PF16905	GPHH	Voltage-dependent L-type calcium channel, IQ-associated
PF16944	KCH	Fungal potassium channel
PF16972	TipE	Na+ channel auxiliary subunit TipE
PF17523	MPS-4	MinK-related peptide, potassium channel accessory sub-unit protein 4

Table S3. Summary of *L. stagnalis* CNS RNA-seq library metrics before and after reads correction and filtering.

Stage	Library	Pair	Sample Name	% Dups	% GC	Length	No. reads (million)
Pre	AE	1	BYD_AEOSRB_7_1_HJLK5BBXX.12BA015_noribo_clean	66.40%	42%	148 bp	20.8
Post	AE	1	AE_corrected_BYD_AEOSRB_7_1_HJLK5BBXX.12BA015_noribo_clean.cor	70.30%	42%	148 bp	19.9
Pre	AE	2	BYD_AEOSRB_7_2_HJLK5BBXX.12BA015_noribo_clean	61.20%	42%	148 bp	20.8
Post	AE	2	AE_corrected_BYD_AEOSRB_7_2_HJLK5BBXX.12BA015_noribo_clean.cor	65.10%	42%	148 bp	19.9
Pre	AF	1	BYD_AFOSRB_7_1_HJLK5BBXX.12BA016_noribo_clean	65.10%	39%	150 bp	21.9
Post	AF	1	AF_corrected_BYD_AFOSRB_7_1_HJLK5BBXX.12BA016_noribo_clean.cor	69.60%	39%	150 bp	21.1
Pre	AF	2	BYD_AFOSRB_7_2_HJLK5BBXX.12BA016_noribo_clean	58.50%	39%	149 bp	21.9
Post	AF	2	AF_corrected_BYD_AFOSRB_7_2_HJLK5BBXX.12BA016_noribo_clean.cor	62.70%	39%	149 bp	21.1
Pre	AG	1	BYD_AGOSRB_7_1_HJLK5BBXX.12BA017_noribo_clean	63.30%	41%	150 bp	19.6
Post	AG	1	AG_corrected_BYD_AGOSRB_7_1_HJLK5BBXX.12BA017_noribo_clean.cor	67.60%	41%	150 bp	18.8
Pre	AG	2	BYD_AGOSRB_7_2_HJLK5BBXX.12BA017_noribo_clean	57.20%	41%	149 bp	19.6
Post	AG	2	AG_corrected_BYD_AGOSRB_7_2_HJLK5BBXX.12BA017_noribo_clean.cor	61.30%	41%	149 bp	18.8
Pre	AH	1	BYD_AHOSRB_7_1_HJLK5BBXX.12BA018_noribo_clean	68.50%	40%	150 bp	19.5
Post	AH	1	AH_corrected_BYD_AHOSRB_7_1_HJLK5BBXX.12BA018_noribo_clean.cor	72.10%	40%	150 bp	18.7
Pre	AH	2	BYD_AHOSRB_7_2_HJLK5BBXX.12BA018_noribo_clean	61.00%	40%	149 bp	19.5
Post	AH	2	AH_corrected_BYD_AHOSRB_7_2_HJLK5BBXX.12BA018_noribo_clean.cor	66.00%	40%	149 bp	18.7
Pre	DRR	1	DRR002012_1	45.20%	40%	100 bp	81.9
Post	DRR	1	filtered_DRR002012_1.cor	62.50%	40%	100 bp	78.9

Table S4. Mapping statistics of the CNS RNA-seq libraries to *L. stagnalis* genome assembly.

	AF	AH	AE	AG	DRR
**Number of input reads**	21,067,534	18,745,164	19,937,778	18,822,989	78,871,131
**Average input read length**	299 bp	299 bp	296 bp	298 bp	100
**Uniquely mapped reads %**	91.99%	88.18%	89.21%	89.73%	91.63%
**Average mapped length**	296.54	296.43	293.39	296.11	99.43
**Number of splices: Total**	15252015	14483036	16226213	15359849	20071372
**Number of splices: Annotated (sjdb)**	15212903	14433405	16152922	15280173	20019565
**Number of splices: GT/AG**	15085673	14292527	16026483	15156047	19788756
**Number of splices: GC/AG**	123565	121044	137126	144330	161581
**Number of splices: AT/AC**	8633	23118	6928	7401	104349
**Number of splices: Non-canonical**	34144	46347	55676	52071	16686
**Mismatch rate per base, %**	0.43%	0.46%	0.45%	0.46%	0.47%
**Deletion rate per base**	0.06%	0.06%	0.05%	0.05%	0.05%
**Deletion average length**	2.51	2.52	2.58	2.45	2.12
**Insertion rate per base**	0.07%	0.06%	0.05%	0.05%	0.04%
**Insertion average length**	1.61	1.79	1.76	1.74	1.58
**% of reads mapped to multiple loci**	3.60%	5.96%	4.17%	4.44%	5.28%
**% of reads mapped to too many loci**	0.18%	0.19%	0.21%	0.21%	0.40%
**% of reads unmapped: too many mismatches**	0.00%	0.00%	0.00%	0.00%	0.00%
**% of reads unmapped: too short**	3.53%	4.69%	5.31%	4.44%	2.11%
**% of reads unmapped: other**	0.71%	0.99%	1.09%	1.17%	0.58%
**Percentage of chimeric reads**	0%	0%	0%	0%	0.00%

Table S5. Transcript assembly statistics for *L. stagnalis* CNS RNA-seq libraries.

Assembler	RNA-seq library	No. transcripts
Scallop	AE	41,310
	AF	42,669
	AG	42,738
	AH	42,374
	DRR002012	74,333
Strawberry	AE	64,505
	AF	62,723
	AG	65,244
	AH	65,418
	DRR002012	66,499
Stringtie	AE	58,381
	AF	53,015
	AG	54,633
	AH	60,904
	DRR002012	52,342
Trinity (genome-guided)	AE	135,736
	AF	139,718
	AG	143,094
	AH	141,017
	DRR002012	127,595
Trinity (*de novo*)	AE, AF, AG and AH (pooled unmapped reads)	108,107
	DRR2012 (unmapped reads)	9,569
Total		1,651,924

Table S6. Proportion of transcripts containing complete and fragmented ORFs as identified by the Evigene pipeline in the "okay" and "okalt" sequence sets.

	**"Okay" set**	**"Okalt" set**
Complete *	85,137 (87.26%)	71,556 (72.32%)
3' partial (lack stop codon) *	3,809 (3.90%)	11,045 (11.16%)
5' partial (lack start codon)	5,149 (5.28%)	7,509 (7.59%)
Internal (lack start and stop codons)	3,470 (3.56%)	8,839 (8.93%)
Total	**97,565**	**98,949**

* Sequences used for downstream analyses.

Table S7. Top 20 expressed transcripts in the adult mouse brain.

ID	Name	Description
ENSMUST00000082407.1	Mitochondrially encoded ATP synthase 8	Energy production
ENSMUST00000084013.1	NADH-ubiquinone oxidoreductase chain 4L	
ENSMUST00000082409.1	Mitochondrially encoded cytochrome c oxidase III	
ENSMUST00000082402.1	Mitochondrially encoded cytochrome c oxidase I	
ENSMUST00000082408.1	Mitochondrially encoded ATP synthase 6	
ENSMUST00000082392.1	NADH-ubiquinone oxidoreductase chain 1	
ENSMUST00000082405.1	Mitochondrially encoded cytochrome c oxidase II	
ENSMUST00000082421.1	Mitochondrially encoded cytochrome b	
ENSMUST00000194059.1	SNRPN upstream reading frame protein	ATPase binding
ENSMUST00000082396.1	Mitochondrially encoded NADH dehydrogenase 2	Energy production
ENSMUST00000082414.1	NADH-ubiquinone oxidoreductase chain 4	
ENSMUST00000082419.1	NADH-ubiquinone oxidoreductase chain 6	
ENSMUST00000082418.1	Mitochondrially encoded NADH dehydrogenase 5	
ENSMUST00000128482.7	40S ribosomal protein S15a	Protein synthesis
ENSMUST00000133193.7	Myelin basic protein	Myelination
ENSMUST00000025563.6	Ferritin heavy chain, N-terminally processed	Metal ion homeostasis
ENSMUST00000082411.1	NADH-ubiquinone oxidoreductase chain 3	Energy production
ENSMUST00000040440.6	Calmodulin-1	Signal transduction
ENSMUST00000108707.2	Ubiquitin C	Stress response
ENSMUST00000165126.8	Ubiquitin A-52 residue ribosomal protein fusion product 1	Protein synthesis

Table S8. Top 20 expressed transcripts in the adult *X. tropicalis* brain.

ID	Transcript name	Description
rna57878	Mediator complex subunit 27	Protein synthesis
rna28486	Mitochondrial cytochrome c oxidase III	Energy production
rna38736	SRY-box 18	Gene expression
rna31283	Mitochondrial seryl-tRNA synthetase 2	Protein synthesis
rna27571	Thymosin beta 4 X-linked	Cytoskeleton
rna37560	Protein inhibitor of activated STAT 3	Gene expression
rna34047	Dishevelled segment polarity protein 2	Signal transduction
rna47191	FK506 binding protein 1B	
rna961	Eukaryotic translation elongation factor 1 alpha 1	Protein synthesis
rna19929	Cold inducible RNA binding protein	Protein synthesis
rna2442	Actin gamma 1	Cytoskeleton
rna35596	Cytochrome c oxidase subunit 4I2	Energy production
rna63832	Ribosomal protein L39	Protein synthesis
rna11539	Ribosomal protein, large, P1	Protein synthesis
rna3828	Actin, beta	Cytoskeleton
rna16400	Synaptosome associated protein 25kDa	Synaptic transmission
rna625	Peptidylprolyl isomerase A (cyclophilin A)	
rna37099	Acyl-CoA binding domain containing 7	
rna9270	Eukaryotic translation elongation factor 2, gene 1	Protein synthesis
rna14953	Ferritin, heavy polypeptide 1	Metal ion homeostasis

Table S9. Top 20 expressed transcripts in the adult zebrafish brain.

ID	Name	Description
ENSDART00000093611.3	ATP synthase 8, mitochondrial	Energy production
ENSDART00000182970.1	BX548011.2	Unknown
ENSDART00000093609.3	Cytochrome c oxidase II, mitochondrial	Energy production
ENSDART00000093606.3	Cytochrome c oxidase I, mitochondrial	
ENSDART00000093613.3	Cytochrome c oxidase III, mitochondrial	
ENSDART00000093612.3	ATP synthase 6, mitochondrial	
ENSDART00000171617.2	Ependymin	
ENSDART00000188084.1	BX571981.3	Unknown
ENSDART00000181887.1	CR352249.6	Unknown
ENSDART00000093617.3	NADH dehydrogenase 4L, mitochondrial	Energy production
ENSDART00000093625.3	Cytochrome b, mitochondrial	Energy production
ENSDART00000182119.1	BX470076.2	Unknown
ENSDART00000149639.2	Myelin basic protein a	Myelination
ENSDART00000052556.8	Myelin basic protein a	
ENSDART00000182410.1	NADH dehydrogenase (ubiquinone) 1 alpha subcomplex, 4	Energy production
ENSDART00000093615.3	NADH dehydrogenase 3, mitochondrial	
ENSDART00000093623.3	NADH dehydrogenase 6, mitochondrial	
ENSDART00000191912.1	BX005436.3	Unknown
ENSDART00000186782.1	Metallothionein 2	
ENSDART00000186881.1	BX511120.1	Unknown

Table S10. Top 20 expressed transcripts in the adult fruitfly brain.

ID	Name	Description
FBtr0100861	Mitochondrial cytochrome c oxidase subunit I	Energy production
FBtr0100868	Mitochondrial cytochrome c oxidase subunit III	
FBtr0082158	Metallothionein A	Metal ion homeostasis
FBtr0100863	Mitochondrial cytochrome c oxidase subunit II	Energy production
FBtr0433502	Mitochondrial cytochrome b	
FBtr0072185	Ribosomal protein L39	Protein synthesis
FBtr0100231	Ribosomal protein L41	
FBtr0433498	Mitochondrial ATPase subunit 6	Energy production
FBtr0305669	Ribosomal protein L29	Protein synthesis
FBtr0088816	Odorant-binding protein 44a	Sensory perception
FBtr0081920	Bcdna:gh11984	Tissue patterning
FBtr0345321	Ribosomal protein L36	Protein synthesis
FBtr0100870	Mitochondrial NADH-ubiquinone oxidoreductase chain 3	Energy production
FBtr0083970	Ribosomal protein S30	Protein synthesis
FBtr0111120	Ribosomal protein L38	
FBtr0433499	Mitochondrial NADH-ubiquinone oxidoreductase chain 1	Energy production
FBtr0071897	Ribosomal protein L23	Protein synthesis
FBtr0111132	Ribosomal protein L5	
FBtr0082136	Ribosomal protein S29	
FBtr0071360	Ribosomal protein s28b	

Table S11. Top 20 expressed transcripts in adult *C. elegans* neurons.

ID	Name	Description
C26F1.9	60S ribosomal protein L39	Protein synthesis
F46A8.7	Unannotated	
R102.2	Unannotated	
Y37D8A.15.1	FMRFamide-like neuropeptides 14	Neuropeptide signaling
B0513.3a	60S ribosomal protein L29	Protein synthesis
B0513.3b	60S ribosomal protein L29	
Y41D4B.5	40S ribosomal protein S28	
C06B8.8.6	60S ribosomal protein L38	
T27C4.1.2	Unannotated	
M03D4.3	Unannotated	
M142.1a	Unc-119	Signal transduction
C07A12.2	Unannotated	
Y48B6A.2.2	60S ribosomal protein L37a	Protein synthesis
F54D7.7	Ribosomal Protein, Large subunit	
F07D3.2	FMRFamide-like neuropeptides 6	Neuropeptide signaling
D1007.12	60S ribosomal protein L24	Protein synthesis
T03D8.3	Seven B Two homolog	Neuropeptide signaling
T20B3.14	Unannotated	
F02A9.2b	Fatty-acid and retinol-binding protein 1	Lipid binding
F02A9.3.2	Fatty-acid and retinol-binding protein 2	

Table S12. Enriched GO terms of mouse genes in orthogroups shared amongst vertebrate and invertebrate species.

Domain	ID	Term	P-value
Biological process	GO:0044237	Cellular metabolic process	1.57E-189
	GO:0071840	Cellular component organization or biogenesis	3.45E-122
	GO:0051641	Cellular localization	1.47E-107
	GO:0051128	Regulation of cellular component organization	1.44E-46
	GO:0007399	Nervous system development	3.27E-31
	GO:0033554	Cellular response to stress	4.69E-24
	GO:0070887	Cellular response to chemical stimulus	2.31E-16
	GO:0099537	Trans-synaptic signaling	7.15E-15
	GO:0035556	Intracellular signal transduction	1.23E-14
	GO:0071495	Cellular response to endogenous stimulus	5.54E-11
	GO:0040011	Locomotion	2.73E-10
	GO:0007610	Behavior	5.62E-10
	GO:0016032	Viral process	9.79E-08
	GO:0009628	Response to abiotic stimulus	0.000000226
	GO:0007611	Learning or memory	0.00000168
	GO:0040007	Growth	0.0000059
	GO:2000300	Regulation of synaptic vesicle exocytosis	0.0000102
	GO:0072583	Clathrin-dependent endocytosis	0.0000654
	GO:0015012	Heparan sulfate proteoglycan biosynthetic process	0.000214
	GO:0048013	Ephrin receptor signaling pathway	0.000556
	GO:0050885	Neuromuscular process controlling balance	0.000557
	GO:2000463	Positive regulation of excitatory postsynaptic potential	0.00101
	GO:0006936	Muscle contraction	0.00137
	GO:0019751	Polyol metabolic process	0.00371
	GO:0010631	Epithelial cell migration	0.00465
	GO:0032412	Regulation of ion transmembrane transporter activity	0.0123
	GO:0060047	Heart contraction	0.0144
	GO:0007215	Glutamate receptor signaling pathway	0.0147
	GO:1990823	Response to leukemia inhibitory factor	0.0161
	GO:0006096	Glycolytic process	0.0184
	GO:0006821	Chloride transport	0.024
	GO:0042552	Myelination	0.0331
	GO:0030518	Intracellular steroid hormone receptor signaling pathway	0.0331
	GO:0009749	Response to glucose	0.0445
Cellular component	GO:0044444	Cytoplasmic part	1.98E-310
	GO:0019898	Extrinsic component of membrane	0.000000176
	GO:0009898	Cytoplasmic side of plasma membrane	0.00161
	GO:0005746	Mitochondrial respiratory chain	0.00474
	GO:0008328	Ionotropic glutamate receptor complex	0.00519
Molecular functions	GO:0003824	Catalytic activity	1.6E-171
	GO:0022839	Ion gated channel activity	0.000000487
	GO:0003713	Transcription coactivator activity	0.0000135
	GO:0005548	Phospholipid transporter activity	0.0000644
	GO:0035255	Ionotropic glutamate receptor binding	0.000378
	GO:0005216	Ion channel activity	0.00133
	GO:0015108	Chloride transmembrane transporter activity	0.0021
	GO:0031489	Myosin V binding	0.00225
	GO:0061650	Ubiquitin-like protein conjugating enzyme activity	0.0037
	GO:0015297	Antiporter activity	0.0192
	GO:0003697	Single-stranded DNA binding	0.0407
	GO:0061659	Ubiquitin-like protein ligase activity	0.0446

Table S13. Enriched Reactome pathways of mouse genes in orthogroups shared amongst all the species examined.

ID	Term	P-value
REAC:R-MMU-8953854	Metabolism of RNA	4.17E-55
REAC:R-MMU-199991	Membrane Trafficking	1.31E-34
REAC:R-MMU-112315	Transmission across Chemical Synapses	1.47E-24
REAC:R-MMU-72766	Translation	2.27E-19
REAC:R-MMU-3700989	Transcriptional Regulation by TP53	5.32E-17
REAC:R-MMU-1430728	Metabolism	5.09E-15
REAC:R-MMU-3858494	Beta-catenin independent WNT signaling	1.72E-14
REAC:R-MMU-422475	Axon guidance	2.39E-14
REAC:R-MMU-75953	RNA Polymerase II Transcription Initiation	8.84E-11
REAC:R-MMU-187037	Signaling by NTRK1 (TRKA)	7.32E-10
REAC:R-MMU-453279	Mitotic G1-G1/S phases	1.12E-09
REAC:R-MMU-69306	DNA Replication	1.24E-08
REAC:R-MMU-446203	Asparagine N-linked glycosylation	1.76E-08
REAC:R-MMU-5696398	Nucleotide Excision Repair	5.57E-08
REAC:R-MMU-6807070	PTEN Regulation	5.82E-08
REAC:R-MMU-382551	Transport of small molecules	1.60E-07
REAC:R-MMU-2262749	Cellular response to hypoxia	3.55E-07
REAC:R-MMU-5610785	GLI3 is processed to GLI3R by the proteasome	1.24E-06
REAC:R-MMU-5687128	MAPK6/MAPK4 signaling	1.97E-06
REAC:R-MMU-8939902	Regulation of RUNX2 expression and activity	7.25E-06
REAC:R-MMU-111885	Opioid Signalling	7.26E-06
REAC:R-MMU-8854050	FBXL7 down-regulates AURKA during mitotic entry and in early mitosis	2.17E-05
REAC:R-MMU-8939236	RUNX1 regulates transcription of genes involved in differentiation of HSCs	2.25E-05
REAC:R-MMU-4641258	Degradation of DVL	2.53E-05
REAC:R-MMU-69613	p53-Independent G1/S DNA damage checkpoint	3.74E-05
REAC:R-MMU-4641257	Degradation of AXIN	9.97E-05
REAC:R-MMU-8878159	Transcriptional regulation by RUNX3	0.00011
REAC:R-MMU-5607761	Dectin-1 mediated noncanonical NF-kB signaling	0.000123
REAC:R-MMU-5676590	NIK-->noncanonical NF-kB signaling	0.000123
REAC:R-MMU-1168372	Downstream signaling events of B Cell Receptor (BCR)	0.000177
REAC:R-MMU-1236978	Cross-presentation of soluble exogenous antigens (endosomes)	0.000304
REAC:R-MMU-1483257	Phospholipid metabolism	0.000931
REAC:R-MMU-68882	Mitotic Anaphase	0.00195
REAC:R-MMU-450282	MAPK targets/ Nuclear events mediated by MAP kinases	0.00303
REAC:R-MMU-8956320	Nucleobase biosynthesis	0.00313
REAC:R-MMU-8852276	The role of GTSE1 in G2/M progression after G2 checkpoint	0.00328
REAC:R-MMU-5621481	C-type lectin receptors (CLRs)	0.00408
REAC:R-MMU-73762	RNA Polymerase I Transcription Initiation	0.0048
REAC:R-MMU-1296059	G protein gated Potassium channels	0.00491
REAC:R-MMU-110320	Translesion Synthesis by POLH	0.00504
REAC:R-MMU-416482	G alpha (12/13) signalling events	0.00683
REAC:R-MMU-8873719	RAB geranylgeranylation	0.0102
REAC:R-MMU-194315	Signaling by Rho GTPases	0.0137
REAC:R-MMU-165159	mTOR signalling	0.0152
REAC:R-MMU-975871	MyD88 cascade initiated on plasma membrane	0.0229
REAC:R-MMU-202424	Downstream TCR signaling	0.0256
REAC:R-MMU-881907	Gastrin-CREB signalling pathway via PKC and MAPK	0.0269
REAC:R-MMU-166058	MyD88:Mal cascade initiated on plasma membrane	0.0355
REAC:R-MMU-418217	G beta:gamma signalling through PLC beta	0.0383
REAC:R-MMU-168138	Toll Like Receptor 9 (TLR9) Cascade	0.043

Table S14. Enriched KEGG pathways of mouse genes in orthogroups shared amongst all the species examined.

ID	Term	P-value
KEGG:03040	Spliceosome	7.71E-26
KEGG:04725	Cholinergic synapse	1.2E-18
KEGG:04728	Dopaminergic synapse	5.09E-18
KEGG:03010	Ribosome	7.98E-18
KEGG:01100	Metabolic pathways	4.27E-17
KEGG:04921	Oxytocin signaling pathway	5.37E-16
KEGG:04144	Endocytosis	1.66E-14
KEGG:04070	Phosphatidylinositol signaling system	2.09E-14
KEGG:03008	Ribosome biogenesis in eukaryotes	2.81E-14
KEGG:03015	mRNA surveillance pathway	3.44E-14
KEGG:04141	Protein processing in endoplasmic reticulum	9.22E-14
KEGG:04720	Long-term potentiation	1.21E-12
KEGG:04261	Adrenergic signaling in cardiomyocytes	9.33E-12
KEGG:04152	AMPK signaling pathway	1.24E-11
KEGG:04713	Circadian entrainment	2.68E-11
KEGG:05211	Renal cell carcinoma	3.21E-11
KEGG:04714	Thermogenesis	3.93E-11
KEGG:04024	cAMP signaling pathway	4.12E-11
KEGG:04910	Insulin signaling pathway	9.44E-11
KEGG:04724	Glutamatergic synapse	1.48E-09
KEGG:04371	Apelin signaling pathway	1.65E-09
KEGG:04723	Retrograde endocannabinoid signaling	2.11E-09
KEGG:04925	Aldosterone synthesis and secretion	3.05E-09
KEGG:04140	Autophagy - animal	5.19E-09
KEGG:04071	Sphingolipid signaling pathway	7.16E-09
KEGG:04012	ErbB signaling pathway	9.43E-09
KEGG:04150	mTOR signaling pathway	1.21E-08
KEGG:05010	Alzheimer's disease	3.31E-08
KEGG:05205	Proteoglycans in cancer	3.94E-08
KEGG:01200	Carbon metabolism	4.05E-08
KEGG:05231	Choline metabolism in cancer	4.41E-08
KEGG:05016	Huntington's disease	4.6E-08
KEGG:04926	Relaxin signaling pathway	5.03E-08
KEGG:04810	Regulation of actin cytoskeleton	5.55E-08
KEGG:04916	Melanogenesis	8.66E-08
KEGG:04922	Glucagon signaling pathway	1.57E-07
KEGG:04914	Progesterone-mediated oocyte maturation	3.86E-07
KEGG:04114	Oocyte meiosis	3.96E-07
KEGG:04721	Synaptic vesicle cycle	0.0000004
KEGG:04934	Cushing's syndrome	4.11E-07
KEGG:00970	Aminoacyl-tRNA biosynthesis	4.27E-07
KEGG:05031	Amphetamine addiction	8.37E-07
KEGG:03013	RNA transport	0.00000101
KEGG:04213	Longevity regulating pathway - multiple species	0.00000117
KEGG:04310	Wnt signaling pathway	0.00000118
KEGG:04727	GABAergic synapse	0.00000119
KEGG:04020	Calcium signaling pathway	0.00000123
KEGG:04360	Axon guidance	0.00000135
KEGG:04931	Insulin resistance	0.00000199
KEGG:05100	Bacterial invasion of epithelial cells	0.00000235
KEGG:04510	Focal adhesion	0.00000454

Table S14 (continued). Enriched KEGG pathways of mouse genes in orthogroups shared amongst all the species examined.

ID	Term	P-value
KEGG:05032	Morphine addiction	0.00000475
KEGG:01230	Biosynthesis of amino acids	0.00000527
KEGG:04136	Autophagy - other	0.00000538
KEGG:04022	cGMP-PKG signaling pathway	0.00000584
KEGG:05213	Endometrial cancer	0.00000732
KEGG:04390	Hippo signaling pathway	0.0000112
KEGG:00020	Citrate cycle (TCA cycle)	0.0000121
KEGG:05210	Colorectal cancer	0.0000141
KEGG:04722	Neurotrophin signaling pathway	0.000016
KEGG:04971	Gastric acid secretion	0.0000175
KEGG:04911	Insulin secretion	0.0000238
KEGG:00562	Inositol phosphate metabolism	0.0000296
KEGG:04730	Long-term depression	0.0000349
KEGG:04666	Fc gamma R-mediated phagocytosis	0.0000425
KEGG:04912	GnRH signaling pathway	0.0000435
KEGG:04211	Longevity regulating pathway	0.0000443
KEGG:05225	Hepatocellular carcinoma	0.0000557
KEGG:05214	Glioma	0.0000569
KEGG:04370	VEGF signaling pathway	0.0000654
KEGG:03420	Nucleotide excision repair	0.0000869
KEGG:03022	Basal transcription factors	0.0000869
KEGG:05412	Arrhythmogenic right ventricular cardiomyopathy (ARVC)	0.000106
KEGG:04072	Phospholipase D signaling pathway	0.000108
KEGG:04550	Signaling pathways regulating pluripotency of stem cells	0.000126
KEGG:04530	Tight junction	0.000154
KEGG:04962	Vasopressin-regulated water reabsorption	0.000173
KEGG:04919	Thyroid hormone signaling pathway	0.000179
KEGG:05012	Parkinson's disease	0.000181
KEGG:04270	Vascular smooth muscle contraction	0.000355
KEGG:04726	Serotonergic synapse	0.000508
KEGG:00240	Pyrimidine metabolism	0.000546
KEGG:04611	Platelet activation	0.000573
KEGG:00510	N-Glycan biosynthesis	0.000585
KEGG:04540	Gap junction	0.000603
KEGG:03018	RNA degradation	0.000607
KEGG:00310	Lysine degradation	0.000794
KEGG:03020	RNA polymerase	0.000885
KEGG:03030	DNA replication	0.000919
KEGG:04932	Non-alcoholic fatty liver disease (NAFLD)	0.00105
KEGG:03050	Proteasome	0.00184
KEGG:00230	Purine metabolism	0.00351
KEGG:04066	HIF-1 signaling pathway	0.00452
KEGG:04110	Cell cycle	0.00453
KEGG:04520	Adherens junction	0.00471
KEGG:00534	Glycosaminoglycan biosynthesis - heparan sulfate / heparin	0.00609
KEGG:04260	Cardiac muscle contraction	0.00693
KEGG:04970	Salivary secretion	0.00729
KEGG:00190	Oxidative phosphorylation	0.0073
KEGG:04915	Estrogen signaling pathway	0.0101
KEGG:04664	Fc epsilon RI signaling pathway	0.0131
KEGG:03060	Protein export	0.0197

Table S14 (continued). Enriched KEGG pathways of mouse genes in orthogroups shared amongst all the species examined.

ID	Term	P-value
KEGG:04015	Rap1 signaling pathway	0.0233
KEGG:04120	Ubiquitin mediated proteolysis	0.0255
KEGG:00564	Glycerophospholipid metabolism	0.0281
KEGG:05220	Chronic myeloid leukemia	0.0285
KEGG:00410	beta-Alanine metabolism	0.0287
KEGG:04924	Renin secretion	0.0316
KEGG:05414	Dilated cardiomyopathy (DCM)	0.0325
KEGG:04927	Cortisol synthesis and secretion	0.0336
KEGG:05230	Central carbon metabolism in cancer	0.0345
KEGG:05410	Hypertrophic cardiomyopathy (HCM)	0.0357
KEGG:00520	Amino sugar and nucleotide sugar metabolism	0.0386
KEGG:01210	2-Oxocarboxylic acid metabolism	0.0426

Table S15. Transcript factors whose binding motifs are enriched in the set of mouse genes in orthogroups shared amongst all the species examined.

ID	Transcription factor	P-value
TF:M01240	BEN	0
TF:M00333	ZF5	5.3E-305
TF:M00803	E2F	5.3E-305
TF:M04662	FOXN4	5.3E-305
TF:M02089	E2F-3	4.6E-280
TF:M01981	ELK-1	2.36E-235
TF:M07380	E2F-4	2.18E-226
TF:M07250	E2F-1	7.55E-214
TF:M00196	Sp1	4.06E-208
TF:M01991	PEA3	9.23E-195
TF:M01660	GABP-alpha	1.72E-194
TF:M08995	CTCF	5.49E-194
TF:M01199	RNF96	3.5E-188
TF:M02874	IRF6	3.57E-179
TF:M02844	BCL6B	1.05E-175
TF:M01993	TEL1	1.44E-174
TF:M07991	Hes1	1.34E-168
TF:M05494	Egr-2	1.31E-163
TF:M02872	IRF4	2.96E-161
TF:M01783	SP2	1.65E-159
TF:M02036	WT1	2.91E-159
TF:M00189	AP-2	1.72E-158
TF:M03876	Kaiso	4.43E-157
TF:M05547	ZAC	3.94E-156
TF:M01104	MOVO-B	1.72E-155
TF:M01986	c-ets-1	1.06E-153
TF:M01989	Ets2	3.74E-152
TF:M05455	SP6	4.76E-142
TF:M05301	CPBP	4.76E-142
TF:M05391	LKLF	4.76E-142
TF:M05665	Sp3	4.76E-142
TF:M02913	SP100	1.54E-140
TF:M01752	ERG	9E-140
TF:M00144	Pax-5	2.85E-139
TF:M01979	ELF4	2.67E-134
TF:M00982	KROX	1.49E-132
TF:M07277	BTEB2	2.49E-126
TF:M02039	GABPalpha	1.41E-121
TF:M07354	Egr-1	1.45E-119
TF:M01992	Erm	1.09E-118
TF:M01984	ERF	4.5E-118
TF:M08867	AP2	1.48E-115
TF:M00976	AhR,	8.95E-112
TF:M05407	Klf17	6.83E-111
TF:M01588	GKLF	4.86E-107
TF:M01857	AP-2alpha	7.05E-107
TF:M01598	ZBED6	1.03E-101
TF:M01988	ER71	1.82E-99
TF:M08005	Tcfl5	1.45E-98
TF:M08878	EGR	4.18E-98

Table S15 (continued). Transcript factors whose binding motifs are enriched in the set of mouse genes in orthogroups shared amongst all the species examined.

ID	Transcription factor	P-value
TF:M01770	XBP-1	1.61E-94
TF:M07415	Elf-1	1.9E-94
TF:M02773	Klf7	1.31E-92
TF:M05541	Zfp536	8.4E-90
TF:M00245	Egr-3	1.09E-88
TF:M00470	AP-2gamma	2.1E-88
TF:M01977	ESE-1	6.82E-88
TF:M00327	Pax-3	3.87E-87
TF:M01987	ER81	2.44E-86
TF:M08874	E2F1	2.13E-85
TF:M01858	AP-2beta	1.04E-84
TF:M02835	Zic1	3.96E-84
TF:M07052	NRF-1	5.96E-84
TF:M07980	Atf-1	1.89E-83
TF:M00981	CREB	1.39E-82
TF:M02745	Ehf	1.27E-81
TF:M07329	Osx	2.29E-81
TF:M01820	CREM	3.46E-76
TF:M00237	AhR	8.85E-76
TF:M03791	GABPalpha_GABPbeta	1.87E-75
TF:M07397	ZBP89	1.56E-74
TF:M02742	E2F-2	2.9E-74
TF:M09685	Fli-1	2.09E-73
TF:M00378	Pax-4	5.03E-73
TF:M01593	Zfx	2.11E-71
TF:M02037	Pet-1	1.59E-70
TF:M01253	CNOT3	1.81E-70
TF:M02744	Egr1	1.76E-68
TF:M00322	c-Myc	2.14E-68
TF:M01990	ETV3	5.35E-66
TF:M02810	Sp4	2.54E-62
TF:M00179	ATF2	1.65E-59
TF:M00178	CREB	2.04E-59
TF:M00114	Tax/CREB	2.31E-59
TF:M02011	HES-1	8.62E-59
TF:M00244	NGFI-C	3.37E-58
TF:M01980	Elf5	2.26E-55
TF:M00341	GABP	4.86E-54
TF:M01975	ELF1	7.3E-54
TF:M01160	Kid3	9.5E-54
TF:M01177	SREBP-2	1.2E-52
TF:M01113	CACD	2.09E-52
TF:M02881	MAX	1.11E-51
TF:M01976	nerf	3.22E-50
TF:M02023	MAZ	1.36E-45
TF:M01167	SAP-1a	3.79E-42
TF:M01072	HIC1	1.44E-41
TF:M00450	Zic3	5.67E-41
TF:M07248	CREB1	1.04E-40
TF:M00032	c-Ets-1(p54)	1.17E-40

Table S15 (continued). Transcript factors whose binding motifs are enriched in the set of mouse genes in orthogroups shared amongst all the species examined.

ID	Transcription factor	P-value
TF:M01837	FKLF	2.01E-40
TF:M01865	BTEB3	7.15E-40
TF:M01047	AP-2alphaA	4.1E-39
TF:M01175	CKROX	3.47E-36
TF:M02012	HIF-1alpha	6.5E-36
TF:M03834	N-Myc	1.93E-35
TF:M07312	ATF-2	2.1E-35
TF:M01243	MTF-1	4.45E-33
TF:M01001	DEAF1	4.86E-33
TF:M07313	ATF-3	8.95E-33
TF:M02885	MYF6	2.99E-32
TF:M02836	Zic2	9.92E-31
TF:M01973	PLAG1	3.64E-30
TF:M02042	Spic	7.3E-30
TF:M04617	LRF	4.68E-29
TF:M08885	HAIRYLIKE	2.91E-27
TF:M03944	Zfp740	3.18E-27
TF:M01744	REX1	3.32E-27
TF:M00466	HIF1	5.79E-26
TF:M02786	Plagl1	6.09E-25
TF:M02865	GMEB1	6.39E-25
TF:M00187	USF	1.81E-24
TF:M00444	VDR	2.29E-24
TF:M02044	YY1	2.6E-24
TF:M07996	Mitf	4.99E-23
TF:M02768	Irf-4	1.28E-22
TF:M02925	TCFAP2C	2.75E-22
TF:M00514	ATF4	4.83E-22
TF:M00726	USF2	3.46E-21
TF:M00483	ATF6	6.55E-21
TF:M00539	Arnt	1.2E-20
TF:M02924	TCFAP2B	3.23E-20
TF:M01652	p53	5.04E-20
TF:M02842	ATF1	7.61E-20
TF:M02040	PDEF	1.54E-19
TF:M08868	ARNTLIKE	4.28E-19
TF:M01876	GABP-beta	6.19E-19
TF:M01299	MECP2	1.19E-18
TF:M01721	PUR1	1.58E-18
TF:M00075	GATA-1	5.46E-18
TF:M06840	Zfp516	6.03E-18
TF:M00446	Spz1	1.29E-17
TF:M03572	DEC1	1.34E-17
TF:M00799	Myc	2.09E-17
TF:M03832	Net	2.85E-17
TF:M07461	KLF	1.15E-16
TF:M00257	RREB-1	2.16E-16
TF:M03558	P73	6.65E-16
TF:M07992	Hes2	6.81E-16
TF:M02887	NR2F2	2.86E-15
TF:M09695	Mxi1	3.9E-15
TF:M07041	HDAC1	8.8E-15
TF:M03574	GCMa	6.72E-14

Table S15 (continued). Transcript factors whose binding motifs are enriched in the set of mouse genes in orthogroups shared amongst all the species examined.

ID	Transcription factor	P-value
TF:M02861	GATA-6	4.79E-13
TF:M07067	USF1	8.4E-13
TF:M03871	GLI	1.24E-12
TF:M02831	Zfp281	3.24E-12
TF:M08869	CLOCKBMAL	1.78E-11
TF:M07322	HSF4	3.81E-11
TF:M06457	ZN451	7.2E-11
TF:M07602	CP2	2.22E-10
TF:M01843	DEC2	4.54E-10
TF:M01249	HIF2alpha	6.8E-10
TF:M01587	FPM315	1.38E-09
TF:M01983	SAP1A	1.85E-09
TF:M07356	HIF2A	2.19E-09
TF:M03824	GCMb	2.27E-09
TF:M01252	E2F-6	2.28E-09
TF:M01864	ATF-4	8.87E-09
TF:M01714	KLF15	1.17E-08
TF:M08905	TIEG1	2.63E-08
TF:M02891	RARA	4.74E-08
TF:M01742	Zfp206	8.04E-08
TF:M00220	SREBP-1	8.06E-08
TF:M08884	GCM	0.000000235
TF:M00491	MAZR	0.00000027
TF:M00985	Stra13	0.000000281
TF:M01111	RBP-Jkappa	0.000000649
TF:M02873	IRF-5	0.00000145
TF:M00325	NRSE	0.00000258
TF:M06843	Sall1	0.00000296
TF:M03846	SMAD5	0.00000377
TF:M00947	CP2/LBP-1c/LSF	0.00000401
TF:M08891	NFKAPPAB	0.00000475
TF:M00033	p300	0.00000508
TF:M09705	REST	0.00000824
TF:M00056	myogenin	0.00000826
TF:M01034	Ebox	0.0000124
TF:M04155	Tfap2a	0.0000146
TF:M07611	p54NRB	0.0000224
TF:M03986	Elk3	0.0000276
TF:M06842	Sall3	0.0000334
TF:M00518	PPARalpha	0.0000491
TF:M07260	Ikaros	0.0000825
TF:M00224	STAT1	0.0000879
TF:M00287	NF-Y	0.0000956
TF:M00733	SMAD4	0.000116
TF:M07979	Arntl	0.000227
TF:M03565	slug	0.000275
TF:M07981	Atf3	0.000285
TF:M07392	Pitx3	0.000318
TF:M08835	ZNF515	0.000355
TF:M00720	CAC-binding	0.000391
TF:M02106	NF-YA	0.000517

Table S15 (continued). Transcript factors whose binding motifs are enriched in the set of mouse genes in orthogroups shared amongst all the species examined.

ID	Transcription factor	P-value
TF:M00264	Staf	0.000667
TF:M03553	KLF3	0.00179
TF:M00721	CACCC-binding	0.00183
TF:M00084	MZF-1	0.00263
TF:M08818	KLF8	0.00403
TF:M02811	Spdef	0.00803
TF:M00646	LF-A1	0.0107
TF:M05959	zfp64	0.012
TF:M00281	RFX1	0.0124
TF:M00706	TFII-I	0.0175
TF:M00979	Pax-6	0.0315
TF:M04293	Barhl1	0.0331
TF:M02772	Jdp2	0.0353
TF:M02934	ZFP187	0.0387
TF:M03915	Klf12	0.0417
TF:M04366	Hoxa11	0.0437

Table S16. Enriched GO terms of mouse genes in orthgroups shared amongst only the vertebrate species examined.

Domain	ID	Term	P-value
Biological process	GO:0048731	System development	3.87E-28
	GO:0051239	Regulation of multicellular organismal process	4.52E-24
	GO:0023051	Regulation of signaling	4.69E-15
	GO:0010646	Regulation of cell communication	1.51E-14
	GO:0000902	Cell morphogenesis	2.93E-12
	GO:0022610	Biological adhesion	9.17E-12
	GO:0007166	Cell surface receptor signaling pathway	9.3E-12
	GO:0042127	Regulation of cell proliferation	0.000000898
	GO:0045937	Positive regulation of phosphate metabolic process	0.00000781
	GO:0030595	Leukocyte chemotaxis	0.0000207
	GO:0006954	Inflammatory response	0.0000317
	GO:0043410	Positive regulation of MAPK cascade	0.0000455
	GO:0050714	Positive regulation of protein secretion	0.000175
	GO:0007204	Positive regulation of cytosolic calcium ion concentration	0.000237
	GO:0003013	Circulatory system process	0.00189
	GO:0031532	Actin cytoskeleton reorganization	0.00237
	GO:0030029	Actin filament-based process	0.00258
	GO:0007218	Neuropeptide signaling pathway	0.0101
	GO:0032648	Regulation of interferon-beta production	0.0255
	GO:0098916	Anterograde trans-synaptic signaling	0.0471
Cellular component	GO:0005886	Plasma membrane	6.27E-15
	GO:0005911	Cell-cell junction	0.000000149
	GO:0031012	Extracellular matrix	0.000000516
	GO:0009986	Cell surface	0.0000477
	GO:0031463	Cul3-ring ubiquitin ligase complex	0.0000808
	GO:0043235	Receptor complex	0.000642
	GO:0016327	Apicolateral plasma membrane	0.0447
Molecular function	GO:0005515	Protein binding	3.21E-09
	GO:0005184	Neuropeptide hormone activity	0.000000075
	GO:0005540	Hyaluronic acid binding	0.000608
	GO:0004896	Cytokine receptor activity	0.000635
	GO:0008373	Sialyltransferase activity	0.00335
	GO:0001653	Peptide receptor activity	0.0136
	GO:0008528	G-protein coupled peptide receptor activity	0.0227

Table S17. Enriched Reactome pathways of mouse genes in orthgroups shared amongst only the vertebrate species examined.

ID	Term	P-value
REAC:R-MMU-373076	Class A/1 (Rhodopsin-like receptors)	6.07E-17
REAC:R-MMU-1474244	Extracellular matrix organization	0.00000104
REAC:R-MMU-418990	Adherens junctions interactions	0.0000215
REAC:R-MMU-4085001	Sialic acid metabolism	0.0000939
REAC:R-MMU-381426	Regulation of Insulin-like Growth Factor (IGF) transport and uptake by Insulin-like Growth Factor Binding Proteins (IGFBPs)	0.000462
REAC:R-MMU-162582	Signal Transduction	0.000519
REAC:R-MMU-388844	Receptor-type tyrosine-protein phosphatases	0.000751
REAC:R-MMU-8957275	Post-translational protein phosphorylation	0.00955
REAC:R-MMU-190861	Gap junction assembly	0.0102
REAC:R-MMU-157858	Gap junction trafficking and regulation	0.0184
REAC:R-MMU-418594	G alpha (i) signalling events	0.0203
REAC:R-MMU-433692	Proton-coupled monocarboxylate transport	0.0361
REAC:R-MMU-190377	FGFR2b ligand binding and activation	0.0455
REAC:R-MMU-5223345	Miscellaneous transport and binding events	0.0481

Table S18. Enriched KEGG pathways of mouse genes in orthgroups shared amongst only the vertebrate species examined.

ID	Term	P-value
KEGG:04060	Cytokine-cytokine receptor interaction	1.98E-12
KEGG:04514	Cell adhesion molecules (CAMs)	2.07E-07
KEGG:04064	NF-kappa B signaling pathway	0.0000399
KEGG:04610	Complement and coagulation cascades	0.000122
KEGG:04080	Neuroactive ligand-receptor interaction	0.00023
KEGG:04151	PI3K-Akt signaling pathway	0.000589
KEGG:00604	Glycosphingolipid biosynthesis - ganglio series	0.00439
KEGG:04360	Axon guidance	0.00897
KEGG:04621	NOD-like receptor signaling pathway	0.0365
KEGG:04620	Toll-like receptor signaling pathway	0.0439

Table S19. Enriched GO terms of fruit fly genes in orthgroups shared amongst only the invertebrate species examined.

Domain	ID	Term	P-value
Biological process	GO:0050808	Synapse organization	1.41E-09
	GO:0007606	Sensory perception of chemical stimulus	3.18E-07
	GO:0010496	Intercellular transport	0.00000877
	GO:1901386	Negative regulation of voltage-gated calcium channel activity	0.00147
	GO:0006182	cGMP biosynthetic process	0.013
Cellular component	GO:0044459	Plasma membrane part	6.04E-10
	GO:0005921	Gap junction	0.00000877
	GO:0008074	Guanylate cyclase complex, soluble	0.00883
Molecular function	GO:0001653	Peptide receptor activity	7.79E-08
	GO:0008528	G-protein coupled peptide receptor activity	0.0000316
	GO:0005243	Gap junction channel activity	0.000228
	GO:0004383	Guanylate cyclase activity	0.00883
