# Supplementary material for: Ion channel profiling of the Lymnaea stagnalis ganglia via transcriptome analysis
Source: BMC Genomics. 2021 Jan 6;22:18. doi: 10.1186/s12864-020-07287-2 (PMC7789530; doi:10.1186/s12864-020-07287-2)
Supplement: Supplementary file 2 — Additional file 2: Table S20. Transcripts encoding ionotropic acetylcholine receptors in the L. stagnalis CNS. Table S21. Transcripts encoding ionotropic GABA or glycine receptors in the L. stagnalis CNS. Table S22. Transcripts encoding ionotropic glutamate receptors in the L. stagnalis CNS. Table S23. Transcripts encoding K+ channels in the L. stagnalis CNS. Table S24. Transcripts encoding Ca2+ channels in the L. stagnalis CNS. Table S25. Transcripts encoding Na + channels in the L. stagnalis CNS. Table S26. Transcripts encoding Cl- channels in the L. stagnalis CNS. Table S27. Transcripts encoding cation channels in the L. stagnalis CNS. Table S28. Transcripts encoding TRP channels in the L. stagnalis CNS. [file 12864_2020_7287_MOESM2_ESM.pdf]

Table S20. Transcripts encoding ionotropic acetylcholine receptors in the *L. stagnalis* CNS.

**Sequence ID**	**Nr hit**	**Query length**	**Subject length**	**Percent identity**
evgLocus_FX_18766	XP_013080421.1 PREDICTED: uncharacterized protein LOC106066011 [Biomphalaria glabrata] *	742	631	56.4
evgLocus_FX_17627	ABA60389.1 nicotinic acetylcholine receptor subunit type K [Lymnaea stagnalis]	501	501	100
evgLocus_FX_18804	ABA60390.1 nicotinic acetylcholine receptor subunit type J [Lymnaea stagnalis]	582	550	100
evgLocus_FX_18879	ABA60381.1 nicotinic acetylcholine receptor subunit type B [Lymnaea stagnalis]	527	527	100
evgLocus_FX_20189	ABA60386.1 nicotinic acetylcholine receptor subunit type G [Lymnaea stagnalis]	618	618	100
evgLocus_FX_22234	ABA60387.1 nicotinic acetylcholine receptor subunit type H [Lymnaea stagnalis]	467	450	100
evgLocus_FX_23187	ABA60384.1 nicotinic acetylcholine receptor subunit type E [Lymnaea stagnalis]	538	538	100
evgLocus_FX_27048	ABA60388.1 nicotinic acetylcholine receptor subunit type I [Lymnaea stagnalis]	527	527	100
evgLocus_scallop_AH_33824	ABA60383.1 nicotinic acetylcholine receptor subunit type D [Lymnaea stagnalis]	103	554	100
evgLocus_FX_20255	ABA60382.1 nicotinic acetylcholine receptor subunit type C [Lymnaea stagnalis]	506	506	99.8
evgLocus_FX_21433	ABA60380.1 nicotinic acetylcholine receptor subunit type A [Lymnaea stagnalis]	485	485	99.8
evgLocus_Scallop_AE_39077	ABA60387.1 nicotinic acetylcholine receptor subunit type H [Lymnaea stagnalis]	346	450	99.6
evgLocus_FX_17911	XP_013094583.1 PREDICTED: neuronal acetylcholine receptor subunit beta-4-like [Biomphalaria glabrata]	625	311	88.6
evgLocus_stringtie_AF_41679	ABA60385.1 nicotinic acetylcholine receptor subunit type F [Lymnaea stagnalis]	571	488	86.6
evgLocus_Scallop_AE_34677	XP_013067706.1 PREDICTED: neuronal acetylcholine receptor subunit beta-3-like [Biomphalaria glabrata]	469	499	82.9
evgLocus_strawberry_AF_33628	XP_005093258.1 PREDICTED: neuronal acetylcholine receptor subunit alpha-7-like [Aplysia californica]	567	468	78.6
evgLocus_FX_21891	XP_013088338.1 PREDICTED: neuronal acetylcholine receptor subunit alpha-2-like [Biomphalaria glabrata]	509	509	75.1
evgLocus_FX_19484	NP_001297423.1 neuronal acetylcholine receptor subunit alpha-10-like precursor [Aplysia californica]	538	541	72.9
evgLocus_FX_19956	XP_005094753.2 PREDICTED: neuronal acetylcholine receptor subunit beta-3-like [Aplysia californica]	499	509	69.2
evgLocus_FX_16787	NP_001297424.1 acetylcholine receptor subunit alpha-type acr-16-like precursor [Aplysia californica]	604	635	68
evgLocus_FX_18755	ABA60390.1 nicotinic acetylcholine receptor subunit type J [Lymnaea stagnalis]	544	550	67.4
evgLocus_FX_21106	NP_001267733.1 acetylcholine receptor subunit beta-like 1-like [Aplysia californica]	587	593	65.3
evgLocus_FX_20080	XP_013090302.1 PREDICTED: neuronal acetylcholine receptor subunit beta-3-like [Biomphalaria glabrata]	581	567	63.9
evgLocus_strawberry_AG_62696	XP_021342101.1 neuronal acetylcholine receptor subunit alpha-10-like [Mizuhopecten yessoensis]	317	482	63.6
evgLocus_FX_19442	XP_021342101.1 neuronal acetylcholine receptor subunit alpha-10-like [Mizuhopecten yessoensis]	487	482	61.1
evgLocus_Scallop_AE_39871	XP_013094335.1 PREDICTED: neuronal acetylcholine receptor subunit beta-3-like [Biomphalaria glabrata]	279	418	60.7
evgLocus_stringtie_AH_15026	XP_013060683.1 PREDICTED: acetylcholine receptor subunit alpha-like [Biomphalaria glabrata]	320	437	55.3
evgLocus_strawberry_AG_33123	XP_021351690.1 neuronal acetylcholine receptor subunit alpha-10-like, partial [Mizuhopecten yessoensis]	620	637	52.8
evgLocus_stringtie_AH_58757	XP_013094335.1 PREDICTED: neuronal acetylcholine receptor subunit beta-3-like [Biomphalaria glabrata]	333	418	51.2
evgLocus_Trinity_AG_42558	XP_013066091.1 PREDICTED: acetylcholine receptor subunit alpha-like [Biomphalaria glabrata]	311	447	50.8
evgLocus_FX_25160	XP_013071749.1 PREDICTED: neuronal acetylcholine receptor subunit alpha-7-like [Biomphalaria glabrata]	449	423	50.4
evgLocus_Trinity_AF_41395	XP_013066091.1 PREDICTED: acetylcholine receptor subunit alpha-like [Biomphalaria glabrata]	460	447	50.4
evgLocus_FX_24558	XP_013066091.1 PREDICTED: acetylcholine receptor subunit alpha-like [Biomphalaria glabrata]	432	447	49.4
evgLocus_FX_22924	XP_013071749.1 PREDICTED: neuronal acetylcholine receptor subunit alpha-7-like [Biomphalaria glabrata]	407	423	46.8
evgLocus_stringtie_AE_5950	XP_012940025.1 PREDICTED: acetylcholine receptor subunit alpha-like [Aplysia californica]	269	397	42
evgLocus_Trinity_AG_60798	NP_001267746.1 ligand-gated ion channel 4-like precursor [Aplysia californica] *	521	518	83.1

* Contains InterPro signature of ionotropic acetylcholine receptor.

Table S21. Transcripts encoding ionotropic GABA or glycine receptors in the *L. stagnalis* CNS.

Sequence ID	Nr hit	Query length	Subject length	Percent identity
evgLocus_FX_19329	CAA50492.1 gamma-aminobutyric acid-grated chloride-ion channel/receptor, zeta subunit [Lymnaea stagnalis]	437	437	100
evgLocus_FX_21251	P26714.1 RecName: Full=Gamma-aminobutyric acid receptor subunit beta	529	499	99.4
evgLocus_FX_20044	XP_013091826.1 PREDICTED: gamma-aminobutyric acid receptor alpha-like isoform X1 [Biomphalaria glabrata]	443	488	90.2
evgLocus_Trinity_AE_79821	XP_013063333.1 PREDICTED: gamma-aminobutyric acid receptor subunit alpha-6-like [Biomphalaria glabrata]	711	706	84.2
evgLocus_FX_26671	XP_013094258.1 PREDICTED: gamma-aminobutyric acid receptor subunit alpha-2-like [Biomphalaria glabrata]	459	447	72.4
evgLocus_FX_25484	NP_001191520.1 GluClAc2 [Aplysia californica]	425	429	79.4
evgLocus_FX_23008	XP_013092685.1 PREDICTED: glycine receptor subunit alpha-3-like isoform X1 [Biomphalaria glabrata]	425	420	74.6
evgLocus_FX_28954	XP_021342615.1 glycine receptor subunit alphaZ1-like [Mizuhopecten yessoensis]	412	387	47.6

Table S22. Transcripts encoding ionotropic glutamate receptors in the *L. stagnalis* CNS.

**Sequence ID**	**Nr hit**	**Query length**	**Subject length**	**Percent identity**
evgLocus_FX_16972	XP_012938956.1 PREDICTED: uncharacterized protein LOC101860416 [Aplysia californica] *	1549	1692	55.3
evgLocus_FX_19909	P26591.1 RecName: Full=Glutamate receptor	917	917	99.8
evgLocus_scallop_AF_4621	CAA60854.1 glutamate receptor InvGluR-K1 polypeptide [Lymnaea stagnalis]	953	953	99.7
evgLocus_FX_19001	AAT40576.1 NMDA-type glutamate receptor [Lymnaea stagnalis]	963	963	99.5
evgLocus_Strawberry_DRR_22588	XP_013081818.1 PREDICTED: glutamate receptor ionotropic, NMDA 2B-like [Biomphalaria glabrata]	1336	1569	61.5
evgLocus_Stringtie_DRR_39190	OWF44765.1 Glutamate receptor ionotropic, NMDA 3A [Mizuhopecten yessoensis]	805	1203	61.5
evgLocus_FX_17076	NP_001191485.1 NR2 [Aplysia californica]	1504	1164	58.7
evgLocus_Trinity_GG_DRR_75560	XP_013072954.1 PREDICTED: glutamate receptor 1-like, partial [Biomphalaria glabrata]	979	935	76.9
evgLocus_strawberry_AE_34164	XP_013081052.1 PREDICTED: glutamate receptor ionotropic, kainate 2-like isoform X1 [Biomphalaria glabrata]	852	549	75.3
evgLocus_FX_19753	NP_001191539.1 glutamate receptor subunit protein GluR1 precursor [Aplysia californica]	945	925	75.1
evgLocus_FX_18618	AAP41206.1 glutamate receptor subunit protein GluR4 [Aplysia californica]	899	906	75
evgLocus_strawberry_AF_7153	NP_001191543.1 glutamate receptor subunit protein GluR7 precursor [Aplysia californica]	899	890	74.9
evgLocus_Trinity_RF_Nov18_27402	XP_013092521.1 PREDICTED: glutamate receptor ionotropic, kainate 2-like [Biomphalaria glabrata]	858	780	74.9
evgLocus_Trinity_AE_24441	XP_012945575.1 PREDICTED: glutamate receptor 3-like [Aplysia californica]	517	407	74.8
evgLocus_strawberry_AF_15779	NP_001191540.1 glutamate receptor subunit protein GluR2 precursor [Aplysia californica]	953	911	72.2
evgLocus_Trinity_AF_81772	NP_001191542.1 glutamate receptor subunit protein GluR6 [Aplysia californica]	768	875	66.9
evgLocus_FX_17854	NP_001191542.1 glutamate receptor subunit protein GluR6 [Aplysia californica]	851	875	63.8
evgLocus_Trinity_AG_83842	NP_001191542.1 glutamate receptor subunit protein GluR6 [Aplysia californica]	804	875	63.6
evgLocus_FX_18725	XP_005102071.2 PREDICTED: glutamate receptor ionotropic, kainate 3 [Aplysia californica]	521	506	62.8
evgLocus_FX_18690	XP_013077978.1 PREDICTED: glutamate receptor 2-like [Biomphalaria glabrata]	569	449	59.9
evgLocus_stringtie_AE_28436	XP_012940304.1 PREDICTED: glutamate receptor ionotropic, kainate 3-like [Aplysia californica]	1033	985	57.1
evgLocus_FX_20287	XP_005107294.2 PREDICTED: glutamate receptor ionotropic, kainate 4-like [Aplysia californica]	494	501	53.4
evgLocus_Trinity_AG_4349	NP_001191541.1 glutamate receptor subunit protein GluR5 precursor [Aplysia californica]	618	903	49.8
evgLocus_scallop_AG_32790	NP_001191541.1 glutamate receptor subunit protein GluR5 precursor [Aplysia californica]	913	903	47.5
evgLocus_Trinity_RF_Nov18_20632	OWF49654.1 Glutamate receptor ionotropic, delta-1 [Mizuhopecten yessoensis]	671	852	46.6
evgLocus_FX_18551	XP_021345787.1 glutamate receptor 4-like [Mizuhopecten yessoensis]	876	909	45.5
evgLocus_Trinity_GG_DRR_28841	XP_009064312.1 hypothetical protein LOTGIDRAFT_131225 [Lottia gigantea] *	1516	865	50.2

* Contains InterPro signature of ionotropic glutamate receptor.

Table S23. Transcripts encoding K^+ channels in the *L. stagnalis* CNS.

Sequence ID	Nr hit	Query length	Subject length	Percent identity
evgLocus_stringtie_AG_14914	XP_005093895.1 PREDICTED: uncharacterized protein LOC101849827 [Aplysia californica] [#]	1463	1558	69.3
evgLocus_scallop_AF_13162	XP_005108858.1 PREDICTED: uncharacterized protein LOC101862142 [Aplysia californica] [#]	221	401	35.9
evgLocus_scallop_AF_8899	XP_013095351.1 PREDICTED: uncharacterized protein LOC106078854 [Biomphalaria glabrata] [§]	1474	1360	68
evgLocus_strawberry_AE_56499	XP_013068853.1 PREDICTED: calcium-activated potassium channel slowpoke-like isoform X9 [Biomphalaria glabrata]	1070	1099	91.9
evgLocus_Strawberry_DRR_58351	XP_013068787.1 PREDICTED: calcium-activated potassium channel slowpoke-like isoform X1 [Biomphalaria glabrata]	1122	1122	91
evgLocus_Trinity_AF_124854	XP_013090634.1 PREDICTED: small conductance calcium-activated potassium channel protein-like [Biomphalaria glabrata]	825	793	80.8
evgLocus_scallop_AF_38033	XP_013090634.1 PREDICTED: small conductance calcium-activated potassium channel protein-like [Biomphalaria glabrata]	801	793	79.8
evgLocus_strawberry_AG_22928	XP_005107423.2 PREDICTED: small conductance calcium-activated potassium channel protein 3-like [Aplysia californica]	204	571	79.2
evgLocus_strawberry_AE_762	XP_005090058.2 PREDICTED: small conductance calcium-activated potassium channel protein-like [Aplysia californica]	475	519	75.5
evgLocus_strawberry_AE_39140	XP_013083299.1 PREDICTED: TWiK family of potassium channels protein 18-like [Biomphalaria glabrata]	563	546	68.5
evgLocus_scallop_AG_37588	ABR37307.1 TASK two-pore domain potassium channel [Lymnaea stagnalis]	364	361	100
evgLocus_FX_24354	XP_009057266.1 hypothetical protein LOTGIDRAFT_121567 [Lottia gigantea] *	465	274	48.7
evgLocus_Trinity_AE_27410	XP_009057266.1 hypothetical protein LOTGIDRAFT_121567 [Lottia gigantea] *	488	274	45.2
evgLocus_Trinity_GG_DRR_75469	NP_001191546.1 Shaw potassium channel Kv3.1a [Aplysia californica]	614	514	93.3
evgLocus_strawberry_AH_31305	XP_013080373.1 PREDICTED: potassium voltage-gated channel protein Shaw-like [Biomphalaria glabrata]	188	697	91
evgLocus_scallop_AG_2744	XP_013063673.1 PREDICTED: G protein-activated inward rectifier potassium channel 4-like isoform X2 [Biomphalaria glabrata]	486	493	89.7
evgLocus_FX_17791	XP_012942106.1 PREDICTED: potassium voltage-gated channel subfamily KQT member 1-like [Aplysia californica]	665	560	88.6
evgLocus_Scallop_AE_33310	XP_013076045.1 PREDICTED: potassium voltage-gated channel protein Shaw-like [Biomphalaria glabrata]	419	571	86.9
evgLocus_FX_17983	XP_013086501.1 PREDICTED: potassium channel subfamily T member 2-like [Biomphalaria glabrata]	1128	1030	86.1
evgLocus_Stringtie_DRR_22702	XP_012934690.1 PREDICTED: potassium voltage-gated channel subfamily KQT member 1-like [Aplysia californica]	533	623	85.1
evgLocus_scallop_AH_32316	XP_005094519.1 PREDICTED: potassium channel subfamily K member 2-like [Aplysia californica]	420	423	83.7
evgLocus_FX_17005	XP_012939351.1 PREDICTED: potassium channel subfamily K member 1-like [Aplysia californica]	348	339	76.3
evgLocus_Trinity_AG_130536	XP_013067170.1 PREDICTED: potassium channel subfamily K member 4-like [Biomphalaria glabrata]	381	352	69
evgLocus_FX_17605	XP_013094150.1 PREDICTED: potassium voltage-gated channel subfamily H member 2-like [Biomphalaria glabrata]	1111	663	83.5
evgLocus_FX_21655	XP_005091742.1 PREDICTED: potassium voltage-gated channel protein Shal-like [Aplysia californica]	689	717	80.3
evgLocus_FX_19780	XP_012944327.1 PREDICTED: potassium channel subfamily T member 2 [Aplysia californica]	1028	1234	79.3
evgLocus_strawberry_AH_21570	XP_013072553.1 PREDICTED: potassium channel subfamily T member 2-like [Biomphalaria glabrata]	1277	1267	72.3
evgLocus_FX_28708	XP_013064952.1 PREDICTED: ATP-sensitive inward rectifier potassium channel 1-like [Biomphalaria glabrata]	453	430	77.9
evgLocus_FX_21494	XP_013080109.1 PREDICTED: potassium voltage-gated channel protein Shaw-like [Biomphalaria glabrata]	667	674	73.9
evgLocus_strawberry_AG_44481	XP_012944008.1 PREDICTED: ATP-sensitive inward rectifier potassium channel 12-like [Aplysia californica]	436	418	71.5
evgLocus_Stringtie_DRR_27728	XP_013085598.1 PREDICTED: potassium voltage-gated channel protein egl-36-like [Biomphalaria glabrata]	310	817	70.5
evgLocus_stringtie_AF_28143	XP_013085598.1 PREDICTED: potassium voltage-gated channel protein egl-36-like [Biomphalaria glabrata]	356	817	68.5
evgLocus_stringtie_AG_29537	XP_005107164.1 PREDICTED: potassium voltage-gated channel subfamily H member 6-like [Aplysia californica]	1239	1365	67.3
evgLocus_FX_17230	XP_013083954.1 PREDICTED: potassium voltage-gated channel protein Shaw-like isoform X1 [Biomphalaria glabrata]	549	540	66.6
evgLocus_Scallop_DRR_4779	XP_005098785.1 PREDICTED: inward rectifier potassium channel 2-like [Aplysia californica]	576	512	59.3
evgLocus_FX_21050	XP_013091822.1 PREDICTED: potassium channel subfamily K member 9-like [Biomphalaria glabrata]	683	389	51.3

* Contains InterPro signature of TASK channel.
# Contains InterPro signature of voltage-gated K^+ channel.
§ Contains InterPro signature of ELK channel.

Table S24. Transcripts encoding Ca^{2+} channels in the *L. stagnalis* CNS.

**Sequence ID**	**Nr hit**	**Query length**	**Subject length**	**Percent identity**
evgLocus_Scallop_AE_23909	XP_013069789.1 PREDICTED: two pore calcium channel protein 1-like [Biomphalaria glabrata]	817	793	72.5
evgLocus_FX_19019	XP_012943292.1 PREDICTED: two pore calcium channel protein 2-like [Aplysia californica]	818	898	62.2
evgLocus_FX_21501	XP_005089220.2 PREDICTED: two pore calcium channel protein 1-like [Aplysia californica]	827	779	75.6
evgLocus_scallop_AG_24453	XP_013069789.1 PREDICTED: two pore calcium channel protein 1-like [Biomphalaria glabrata]	282	793	67.7
evgLocus_FX_42627	AAO83838.2 voltage-dependent L-type calcium channel alpha-1 subunit isoform a [Lymnaea stagnalis]	207	2078	100
evgLocus_scallop_AG_4605	AAO83838.2 voltage-dependent L-type calcium channel alpha-1 subunit isoform a [Lymnaea stagnalis]	1441	2078	99.7
evgLocus_strawberry_AE_27300	AAO83843.2 voltage-dependent T-type calcium channel alpha-1 subunit isoform A [Lymnaea stagnalis]	2878	2886	98.9
evgLocus_Scallop_AE_17458	AAO83843.2 voltage-dependent T-type calcium channel alpha-1 subunit isoform A [Lymnaea stagnalis]	2891	2886	98.2
evgLocus_Trinity_AH_85872	AAO83843.2 voltage-dependent T-type calcium channel alpha-1 subunit isoform A [Lymnaea stagnalis]	1427	2886	93
evgLocus_strawberry_AG_27619	AAO83843.2 voltage-dependent T-type calcium channel alpha-1 subunit isoform A [Lymnaea stagnalis]	2673	2886	92.6
evgLocus_FX_17353	AAO83841.1 voltage-dependent non-L-type calcium channel alpha-1 subunit isoform A [Lymnaea stagnalis]	1340	2141	96.6
evgLocus_Strawberry_DRR_59689	AAO83841.1 voltage-dependent non-L-type calcium channel alpha-1 subunit isoform A [Lymnaea stagnalis]	1705	2141	99.3
evgLocus_Trinity_GG_DRR_50598	AAO83841.1 voltage-dependent non-L-type calcium channel alpha-1 subunit isoform A [Lymnaea stagnalis]	2196	2141	95
evgLocus_FX_35751	XP_005090384.1 PREDICTED: protein orai-2-like [Aplysia californica]	222	226	85.7
evgLocus_Trinity_GG_DRR_35036	XP_013068048.1 PREDICTED: inositol 1,4,5-trisphosphate receptor type 1-like isoform X7 [Biomphalaria glabrata]	2750	2658	94.5
evgLocus_Stringtie_DRR_40665	XP_013068047.1 PREDICTED: inositol 1,4,5-trisphosphate receptor type 1-like isoform X6 [Biomphalaria glabrata]	2739	2663	93.8
evgLocus_Trinity_AE_111397	XP_012935906.1 PREDICTED: inositol 1,4,5-trisphosphate receptor type 1-like [Aplysia californica]	2956	2978	85
evgLocus_Trinity_AE_111398	XP_012935906.1 PREDICTED: inositol 1,4,5-trisphosphate receptor type 1-like [Aplysia californica]	2980	2978	84.8
evgLocus_strawberry_AG_55149	XP_005096332.1 PREDICTED: inositol 1,4,5-trisphosphate receptor type 3-like [Aplysia californica]	1882	2879	78.1
evgLocus_Trinity_AH_95415	XP_009059256.1 hypothetical protein LOTGIDRAFT_123762 [Lottia gigantea] *	5044	5045	67.3
evgLocus_Trinity_GG_DRR_44867	XP_013065477.1 PREDICTED: ryanodine receptor 44F-like [Biomphalaria glabrata]	5238	4926	79
evgLocus_Trinity_AG_97056	XP_013065477.1 PREDICTED: ryanodine receptor 44F-like [Biomphalaria glabrata]	5217	4926	78.9
evgLocus_FX_16361	XP_013065477.1 PREDICTED: ryanodine receptor 44F-like [Biomphalaria glabrata]	5293	4926	77.9
evgLocus_Trinity_GG_DRR_44870	XP_013065477.1 PREDICTED: ryanodine receptor 44F-like [Biomphalaria glabrata]	2015	4926	77.5

* Contains InterPro signature of ryanodine receptor.

Table S25. Transcripts encoding Na^+ channels in the *L. stagnalis* CNS.

Sequence ID	Nr hit	Query length	Subject length	Percent identity
evgLocus_Trinity_AG_31670	AAK20896.1 FMRFamide-gated and pH-modulated sodium channel [Lymnaea stagnalis]	632	632	100
evgLocus_Trinity_GG_DRR_56848	AAK20896.1 FMRFamide-gated and pH-modulated sodium channel [Lymnaea stagnalis]	615	632	90
evgLocus_strawberry_AF_2915	AAF80601.1 FMRFamide-gated Na+ channel [Planorbella trivolvis]	616	622	72.2
evgLocus_Trinity_GG_DRR_14950	AKH03688.1 voltage-gated sodium channel alpha subunit LNav14b21+ [Lymnaea stagnalis]	2011	2006	99.8
evgLocus_strawberry_AE_20077	AKH03687.1 voltage-gated sodium channel alpha subunit LNav14a21+ [Lymnaea stagnalis]	1653	2007	88.6
evgLocus_Scallop_DRR_41110	XP_013076160.1 PREDICTED: amiloride-sensitive sodium channel subunit alpha-like [Biomphalaria glabrata]	550	453	48.4
evgLocus_FX_21168	XP_005095704.2 PREDICTED: amiloride-sensitive sodium channel subunit beta-2-like [Aplysia californica]	487	489	37.3
evgLocus_strawberry_AH_4559	XP_005095704.2 PREDICTED: amiloride-sensitive sodium channel subunit beta-2-like [Aplysia californica]	480	489	35.7
evgLocus_Trinity_AH_110125	XP_009044219.1 hypothetical protein LOTGIDRAFT_228153 [Lottia gigantea] *	621	536	38.1
evgLocus_Trinity_AE_42211	XP_013083408.1 PREDICTED: degenerin del-1-like [Biomphalaria glabrata]	152	313	39.5
evgLocus_Stringtie_DRR_20189	XP_011430312.1 PREDICTED: uncharacterized protein LOC105330372 [Crassostrea gigas] *	347	930	39.4
evgLocus_FX_56442	No match *	134	-1	-1

* Contains InterPro signature of epithelial sodium channel

Table S26. Transcripts encoding Cl^- channels in the *L. stagnalis* CNS.

**Sequence ID**	**Nr hit**	**Query length**	**Subject length**	**Percent identity**
evgLocus_Trinity_AG_17389	XP_013082752.1 PREDICTED: anoctamin-7-like, partial [Biomphalaria glabrata]	1067	862	85.3
evgLocus_FX_17795	XP_013082752.1 PREDICTED: anoctamin-7-like, partial [Biomphalaria glabrata]	871	862	82.9
evgLocus_Trinity_AF_68258	XP_013082999.1 PREDICTED: anoctamin-1-like isoform X5 [Biomphalaria glabrata]	948	950	81.3
evgLocus_Trinity_GG_DRR_87780	XP_013070323.1 PREDICTED: anoctamin-4-like isoform X6 [Biomphalaria glabrata]	921	1022	78.9
evgLocus_Scallop_AE_2473	XP_005096667.1 PREDICTED: anoctamin-10-like isoform X3 [Aplysia californica]	738	703	72.7
evgLocus_stringtie_AH_5459	XP_013070321.1 PREDICTED: anoctamin-4-like isoform X4 [Biomphalaria glabrata]	1038	1037	70.6
evgLocus_Trinity_AH_48109	XP_013070321.1 PREDICTED: anoctamin-4-like isoform X4 [Biomphalaria glabrata]	532	1037	69.4
evgLocus_FX_16654	XP_013086831.1 PREDICTED: anoctamin-8-like [Biomphalaria glabrata]	1157	1155	67.2
evgLocus_scallop_AH_23138	XP_012939123.1 PREDICTED: bestrophin-3-like, partial [Aplysia californica]	565	365	81.5
evgLocus_strawberry_AF_34562	XP_013073025.1 PREDICTED: bestrophin-2-like [Biomphalaria glabrata]	653	340	78.7
evgLocus_stringtie_AG_1836	XP_013070043.1 PREDICTED: chloride channel protein 2-like [Biomphalaria glabrata]	955	949	75.7
evgLocus_Stringtie_DRR_46452	XP_012944768.1 PREDICTED: chloride channel protein A-like [Aplysia californica]	1421	1425	67.3
evgLocus_scallop_AH_14301	XP_013083884.1 PREDICTED: chloride channel CLIC-like protein 1 [Biomphalaria glabrata]	691	585	41.8
evgLocus_Trinity_RF_Nov18_45322	XP_013083884.1 PREDICTED: chloride channel CLIC-like protein 1 [Biomphalaria glabrata]	603	585	40.8
evgLocus_Trinity_AG_27801	XP_013083884.1 PREDICTED: chloride channel CLIC-like protein 1 [Biomphalaria glabrata]	297	585	38.9
evgLocus_FX_21811	XP_013083884.1 PREDICTED: chloride channel CLIC-like protein 1 [Biomphalaria glabrata]	567	585	38.8
evgLocus_Trinity_AE_85637	XP_013073248.1 PREDICTED: H(+)/Cl(-) exchange transporter 3-like [Biomphalaria glabrata]	843	836	84.6
evgLocus_FX_21027	XP_005096600.1 PREDICTED: H(+)/Cl(-) exchange transporter 7-like [Aplysia californica]	808	816	71.4

Table S27. Transcripts encoding cation channels in the *L. stagnalis* CNS.

Sequence ID	Nr hit	Query length	Subject length	Percent identity
evgLocus_Trinity_AG_6913	XP_005093850.1 PREDICTED: cyclic nucleotide-gated cation channel alpha-3-like isoform X1 [Aplysia californica]	664	669	89.5
evgLocus_Trinity_AG_2741	XP_013063129.1 PREDICTED: cyclic nucleotide-gated cation channel beta-1-like [Biomphalaria glabrata]	1478	541	89.3
evgLocus_scallop_AG_4831	XP_013068727.1 PREDICTED: cyclic nucleotide-gated cation channel beta-1-like [Biomphalaria glabrata]	882	1135	89
evgLocus_strawberry_AE_52253	XP_013063129.1 PREDICTED: cyclic nucleotide-gated cation channel beta-1-like [Biomphalaria glabrata]	1239	541	86.5
evgLocus_FX_40927	XP_013081417.1 PREDICTED: cyclic nucleotide-gated channel rod photoreceptor subunit alpha-like, partial [Biomphalaria glabrata]	225	784	94.2
evgLocus_Trinity_AG_111551	XP_013061334.1 PREDICTED: potassium/sodium hyperpolarization-activated cyclic nucleotide-gated channel 4-like [Biomphalaria glabrata]	1229	1251	75.4
evgLocus_FX_26159	XP_013074477.1 PREDICTED: trimeric intracellular cation channel type B-A-like [Biomphalaria glabrata]	291	291	92.8
evgLocus_Trinity_AH_10150	XP_005102510.1 PREDICTED: trimeric intracellular cation channel type A-like [Aplysia californica]	121	269	67
evgLocus_FX_32684	XP_005102510.1 PREDICTED: trimeric intracellular cation channel type A-like [Aplysia californica]	277	269	64.9
evgLocus_Trinity_AH_67380	AGC13755.1 LNALCN [Lymnaea stagnalis]	1742	1742	99.9
evgLocus_FX_16447	XP_013084437.1 PREDICTED: piezo-type mechanosensitive ion channel component 2-like [Biomphalaria glabrata]	1947	2397	70.9
evgLocus_Trinity_AG_136890	CDJ97773.1 Protein FAM38A [Haemonchus contortus]	213	1730	42.1
evgLocus_Trinity_RF_Nov18_88607	XP_013079313.1 PREDICTED: acid-sensing ion channel 5-like isoform X1 [Biomphalaria glabrata]	552	556	69.8
evgLocus_scallop_AF_10390	XP_013067221.1 PREDICTED: CSC1-like protein 2 isoform X3 [Biomphalaria glabrata]	805	804	89.3
evgLocus_FX_26464	XP_005093106.1 PREDICTED: uncharacterized protein LOC101850633 [Aplysia californica] *	360	339	65.2
evgLocus_FX_33387	XP_005100666.1 PREDICTED: voltage-gated hydrogen channel 1-like [Aplysia californica]	218	281	48.1
evgLocus_Scallop_DRR_19272	XP_013062540.1 PREDICTED: uncharacterized protein LOC106051876 isoform X2 [Biomphalaria glabrata] *	517	485	42.1

* Contains InterPro signature of voltage-gated hydrogen channel.

Table S28. Transcripts encoding TRP channels in the *L. stagnalis* CNS.

Sequence ID	Nr hit	Query length	Subject length	Percent identity
evgLocus_Stringtie_DRR_44113	XP_013074930.1 PREDICTED: uncharacterized protein LOC106061369 [Biomphalaria glabrata] *	460	1247	62.6
evgLocus_strawberry_AH_26500	XP_013074930.1 PREDICTED: uncharacterized protein LOC106061369 [Biomphalaria glabrata] *	610	1247	72.5
evgLocus_Trinity_AF_71664	XP_013083118.1 PREDICTED: transient receptor potential cation channel subfamily V member 5-like [Biomphalaria glabrata]	299	811	70.2
evgLocus_Scallop_AE_1234	XP_011431159.1 PREDICTED: uncharacterized protein LOC105330915 isoform X3 [Crassostrea gigas] *	1037	1120	53.1
evgLocus_stringtie_AH_8895	XP_005101687.1 PREDICTED: uncharacterized protein LOC101851051 [Aplysia californica] *	977	979	52.3
evgLocus_Stringtie_DRR_23400	XP_013094766.1 PREDICTED: transient receptor potential cation channel subfamily A member 1 homolog [Biomphalaria glabrata]	1260	1273	74.5
evgLocus_scallop_AF_8457	XP_012939582.1 PREDICTED: transient receptor potential cation channel subfamily A member 1-like [Aplysia californica]	1271	1264	68.8
evgLocus_Trinity_GG_DRR_1427	XP_012934963.1 PREDICTED: short transient receptor potential channel 4-like [Aplysia californica]	1110	1145	74.5
evgLocus_Trinity_AE_79874	XP_012936952.1 PREDICTED: short transient receptor potential channel 3-like [Aplysia californica]	1033	1047	74.2
evgLocus_Trinity_AH_82800	XP_012936952.1 PREDICTED: short transient receptor potential channel 3-like [Aplysia californica]	559	1047	72.9
evgLocus_FX_18071	XP_012939119.1 PREDICTED: short transient receptor potential channel 7-like [Aplysia californica]	997	779	70
evgLocus_Trinity_AG_2187	XP_012939119.1 PREDICTED: short transient receptor potential channel 7-like [Aplysia californica]	971	779	67.9
evgLocus_FX_18462	XP_012936952.1 PREDICTED: short transient receptor potential channel 3-like [Aplysia californica]	958	1047	64.4
evgLocus_scallop_AH_33484	XP_013076601.1 PREDICTED: short transient receptor potential channel 3-like [Biomphalaria glabrata]	1660	921	83.1
evgLocus_FX_21483	XP_012936952.1 PREDICTED: short transient receptor potential channel 3-like [Aplysia californica]	806	1047	79.4
evgLocus_FX_19171	XP_013080473.1 PREDICTED: short transient receptor potential channel 3-like [Biomphalaria glabrata]	984	1021	48.4
evgLocus_Trinity_AH_49952	XP_021376838.1 transient-receptor-potential-like protein [Mizuhopecten yessoensis]	977	935	63
evgLocus_Trinity_AE_48360	XP_021376838.1 transient-receptor-potential-like protein [Mizuhopecten yessoensis]	952	935	62.1
evgLocus_Scallop_AE_2814	XP_013083410.1 PREDICTED: transient receptor potential cation channel subfamily M member 1-like [Biomphalaria glabrata]	1735	1714	69.3
evgLocus_Trinity_GG_DRR_79297	XP_013083410.1 PREDICTED: transient receptor potential cation channel subfamily M member 1-like [Biomphalaria glabrata]	1840	1714	66
evgLocus_Scallop_DRR_21600	XP_013061400.1 PREDICTED: transient receptor potential cation channel subfamily M member 5-like [Biomphalaria glabrata]	1613	1515	65.4
evgLocus_stringtie_AF_16138	XP_013061400.1 PREDICTED: transient receptor potential cation channel subfamily M member 5-like [Biomphalaria glabrata]	351	1515	61.8
evgLocus_FX_30259	XP_013061400.1 PREDICTED: transient receptor potential cation channel subfamily M member 5-like [Biomphalaria glabrata]	403	1515	59.1
evgLocus_stringtie_AH_11414	XP_013090385.1 PREDICTED: transient receptor potential cation channel subfamily M member 3-like [Biomphalaria glabrata]	509	1426	58.2
evgLocus_Scallop_DRR_73768	XP_013071668.1 PREDICTED: transient receptor potential cation channel subfamily M member 1-like isoform X1 [Biomphalaria glabrata]	672	332	52.8
evgLocus_Trinity_AE_125438	XP_013071668.1 PREDICTED: transient receptor potential cation channel subfamily M member 1-like isoform X1 [Biomphalaria glabrata]	685	332	52.2
evgLocus_Scallop_DRR_44684	XP_013065691.1 PREDICTED: transient receptor potential cation channel subfamily M member 5-like [Biomphalaria glabrata]	1065	877	32.6
evgLocus_Stringtie_DRR_24326	XP_005105591.2 PREDICTED: polycystic kidney disease protein 1-like 2 [Aplysia californica]	583	1857	71.2
evgLocus_Trinity_GG_DRR_24759	XP_005098719.2 PREDICTED: polycystic kidney disease 2-like 1 protein [Aplysia californica]	856	883	85.7
evgLocus_Scallop_DRR_54844	XP_012934947.1 PREDICTED: polycystic kidney disease protein 1-like 2 [Aplysia californica]	1157	1089	60.9
evgLocus_Stringtie_DRR_46096	XP_005088946.2 PREDICTED: polycystic kidney disease protein 1-like 1 [Aplysia californica]	4350	2480	56.9
evgLocus_Trinity_AE_49453	XP_013078401.1 PREDICTED: polycystic kidney disease protein 1-like 2 [Biomphalaria glabrata]	614	1838	50.5
evgLocus_FX_22051	XP_013071763.1 PREDICTED: mucolipin-3-like [Biomphalaria glabrata]	727	592	75.6

* Contains InterPro signature of TRPV channel.
